# The Optimal Lung Cancer Ct Screening Strategy: A Cost‐Effectiveness Study Incorporating Nelson Evidence, Evolving Cost of Treatment and Smoking Cessation Interventions

**DOI:** 10.1002/ijc.70655

**Published:** 2026-07-29

**Authors:** Koen de Nijs, Kevin ten Haaf, Dana Moldovanu, Juul Hubert, Isabelle van den Bosch, Carlijn van der Aalst, Harry J. de Koning

**Affiliations:** ^1^ Department of Public Health Erasmus MC—University Medical Center Rotterdam Rotterdam CE the Netherlands

**Keywords:** cancer early detection, computed tomography, cost‐effectiveness analysis, lung cancer, mass screening

## Abstract

Lung cancer screening implementation is taking shape worldwide. Programme design requires specifying eligibility criteria, screening interval, and the optimal age range. We estimate benefits and harms of competing strategies, integrating NELSON results, smoking cessation interventions, contemporary treatment costs, and the management of feasible CT capacity. 1080 strategies are evaluated using the MISCAN‐Lung microsimulation model, calibrated to NELSON and NLST data. 1945–1979 Dutch cohorts are simulated using a representative smoking history generator. For each strategy, the (cost‐)effectiveness is evaluated, as well as CT requirements and the incremental benefit of a smoking cessation intervention (pharmacotherapy). Lung cancer screening was found to be cost‐effective overall. Risk‐based recruitment (PLCOm2012) is more efficient than pack‐year‐based criteria. Annual screening ages 55–75 from > 1.5% lung cancer risk is cost‐efficient (ICER < €20 k), and would cost €12,201 per quality‐adjusted life‐year (QALY) relative to no screening (or €19,713 compared to less intensive screening). Population‐wide, lung cancer mortality would be reduced by 10.8% (1072 cases/year) at full uptake, or by 6.2% at 50% uptake. For the first three years, at 50% uptake of screening, the strategy requires a 16.1% increase of national CT volume. Nearly half of CT costs are offset by reductions in terminal care expenditures. Integrated smoking cessation is cost‐effective at €10,043/QALY (relative to screening without integrated cessation), yielding 32% additional life‐years gained. Lung cancer screening is increasingly cost‐effective and may be critically considered for implementation, including integrated smoking cessation. We find annual screening ages 55–75 for those > 1.5% (PLCOm) risk to be within a €20 k willingness‐to‐pay and feasible CT requirements.

## What's New?

Lung cancer screening implementation is taking shape worldwide. Programme design, however, requires specifying eligibility criteria, screening interval, and the optimal age range. This modelling study presents a health economic evaluation of over 1000 possible lung screening strategies for the Dutch population, accounting for the required computed tomography capacity, incorporating NELSON‐trial screening effectiveness, and evaluating the incremental cost and benefit of supplementary smoking cessation services. Using the 55–75‐year screening age with a higher than 1.51% PLCOm risk was found to be the optimal strategy at a €20,000 willingness‐to‐pay per quality‐adjusted life‐year, with smoking cessation support a cost‐efficient supplement to screening.

## Graphical abstract

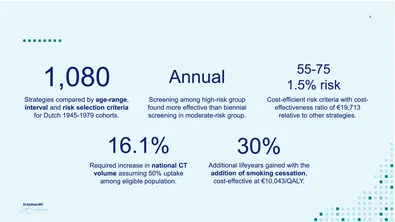

**Abbreviations**
(N)SCLC: (non) small cell lung cancer
ACER: average cost‐effectiveness ratio
CBS: Statistics Netherlands
CHEERS: consolidated health economic evaluation reporting standards
CI: confidence interval
CPD: cigarettes per day
CT: computed tomography
GDP: gross domestic product
ICER: incremental cost‐effectiveness ratio
IKNL: Netherlands Comprehensive Cancer Organisation
LC: lung cancer
LY: life years
MISCAN: Microsimulation Screening ANalysis
NCR: Netherlands Cancer Registry
NELSON: Dutch–Belgian lung cancer screening trial
NLST: National Lung Screening Trial
NMB: net monetary benefit
OR: odds ratio
PLCOm: prostate lung colorectal and ovarian screening trial lung cancer risk model
PY: pack‐years
QALY: quality‐adjusted life years
TLHC: targeted lung health check
TNM: tumour nodule metastasis classification of malignant tumours
USPSTF: United States Preventive Services Task Force
WHO: World Health Organization
WTP: willigness‐to‐pay

## 1 Introduction

Lung cancer is commonly detected in an advanced state, at which point treatment options are limited and survival rates are low [1]. Consequentially, it leads other cancer sites in total deaths, despite being third in total incident cases behind breast‐ and colorectal cancer [2]. In the past decades, age‐adjusted mortality rates have decreased as a consequence of tobacco control and novel treatment pathways [3, 4, 5]. However, with the delayed dose–response relationship between smoking and lung cancer onset, as well as female lung cancer incidence rates and ageing populations, absolute cases of lung cancer are still peaking across the EU [4].

Early‐stage lung cancer can be treated with surgical resection and has better survival outcomes than late‐stage lung cancer. In the Netherlands, Stage I lung cancer has a 5‐year survival of 61%, relative to 7% for Stage IV lung cancer [1]. To improve cancer detection in Stage I, when the disease is generally asymptomatic and unlikely to present clinically, targeted screening of high‐risk ever‐smokers with Computed Tomography (CT) may be used. After demonstrated LC mortality reductions of 20% in the National Lung Screening Trial (NLST) [6] and 24% among males in the Dutch–Belgian lung cancer screening trial (NELSON) [7], many countries are implementing or evaluating structured screening programmes [8]. In the US, the United States Preventive Services Taks Force (USPSTF) recommends annual screening from ages 50–80 for those with at least 20 pack‐years and maximally 15 years since smoking cessation [9]. In the UK, the National Health Service Targeted Lung Health Check (TLHC) offers biennial CT to those with a 1.51% PLCOm risk score between ages 55–74, set for complete roll‐out by 2028–2029 [10].

For mainland Europe, efforts and strategies of screening are scattered. Strategies of risk selection into screening vary, as well as the preferred interval of screening and age range. To illustrate, the Czech Republic has a set‐duration (2022–2026) programme screening ages 55–74 from > 20 pack‐years and with no specified interval, Poland maintains the same eligibility criteria but screens 50–74 years, and Croatia screens from 30 pack‐years between ages 50–75 with personalised screening intervals [11]. Several considerations are of importance when designing a population‐based programme. Since younger individuals have lower lung cancer risk, it is associated with a higher number needed to screen per cancer detected. Similarly, targeted screening among only the highest‐risk group efficiently detects cancers but only among a select group with lower‐than‐average remaining life expectancy. Biennial screening, relative to annual screening, reduces the total burden of screening to the health care system and the participant [12, 13], but incurs a higher risk of interval cancers, cases which generally present in a more advanced stage [7].

Here, we present a health economic evaluation of 1080 possible strategies of screening for the Netherlands, including predictions of lung cancer outcomes and screening‐related harms. The cost‐effectiveness of lung cancer screening has been demonstrated before [14], and other studies have considered optimal screening strategy parameters [13, 15, 16, 17, 18]. However, we present several key contributions to the literature that reflect more accurately the current state of play of lung cancer screening. First, we employ contemporary costs of lung cancer treatment by stage and histology that were purposefully derived from the entire Dutch lung cancer patient population [19]. Treatment costs were found to have increased 52% in 2021 from just 5 years previous. We found novel treatment patterns to have shifted both cost and relative survival of late‐stage cancers, which impact the relative benefit of screening, but also the relative cost as compared to the case‐mix in a no‐screening scenario. Second, we use a microsimulation model of lung cancer natural history and screening effectiveness that was recalibrated to individual‐level data from the NELSON trial [20]. This represents the most recent and representative evidence of lung cancer screening in mainland Europe. Third, we evaluate the relative contribution of supplementary smoking cessation services in the screening context, both in cost and health benefits to the screening participant [21]. Smoking cessation interventions are common practice in lung cancer screening and will impact both the budget requirement and benefits of a screening programme. We compare a range of screening strategies by starting age, stopping age, and risk selection strategy. Strategies are compared by the benefit‐to‐harms ratio of (quality‐adjusted) life years gained and cause‐specific mortality reduction, relative to the monetary cost and the required CT screening capacity incurred.

## 2 Methods

We performed a health economic evaluation of 1080 possible strategies of lung cancer screening for the Netherlands. A microsimulation model of smoking behaviour, non‐lung cancer mortality, and lung cancer natural history was used, with life‐time follow‐up [20]. Individual‐level life histories of individuals from 1949 to 1979 birth cohorts were simulated and aggregated to match the size and composition of the 2023 Dutch population. Health economic assumptions are tallied in the supplied Consolidated Health Economic Evaluation Reporting Standards (CHEERS) checklist [22]. A public payer perspective was used, accounting for costs incurred by the health care sector. We investigated the most efficient strategies in the decision space, that is those that gain the most Quality‐Adjusted Life Years (QALYs) at a given level of cost, as well as the feasibility of each strategy, measured by how much the national CT capacity would need to increase to facilitate uptake. We expect a CT volume increase > 20% to be infeasible.

### 2.1 MISCAN Lung Model

To simulate lung cancer epidemiology under no screening and various screening scenarios, the Microsimulation Screening Analysis (MISCAN) Lung model was used. The model aggregates many repeat simulations of individual constituents of a population to come to population‐level benefits and harms of screening strategies. Smoking initiation, cessation, and number of cigarettes per day were simulated in accordance with self‐reported behaviour in 30 years of the Dutch Health Survey (1989–2020) [21, 23]. From a life history of smoking, lung cancer onset was generated per a validated dose–response model linking cigarette exposure to lung cancer incidence [24]. After lung cancer onset, the cancer progresses from stages IA, IB, II, IIIA, IIIB to Stage IV or is detected in a given stage prior to progression. The period of time spent in each stage of cancer was matched to the stage distribution of lung cancer in the Netherlands and screen‐detected and interval cancer rates in the NLST and NELSON [20]. In an analysis of a screening scenario, screening events may be added to the life history, upon which the cancer may be detected with a stage‐ and histology‐specific sensitivity consistent with individual‐level NELSON outcomes [20]. In the model, if a cancer is detected, it may be cured with a stage‐specific probability of screening effectiveness, calibrated to NELSON rates of cancer mortality [20]. Upon cure, the individual life history will terminate upon their assigned age of death from other causes, rather than the potentially earlier age of death from lung cancer. The aggregate life years saved in this scenario report the population‐level benefits of screening. A more elaborate model description is given in the methodological supplement.

### 2.2 Screening Strategies

We performed 1080 simulations of lung cancer screening, varying in the starting ages, stopping ages, and inclusion criteria of the screening strategies studied. Specifically, we considered:

- Starting ages of 50, 55, and 60.
- Stopping ages of 75, 80 and 85.
- Annual, biennial, and triennial screening.
- Smoking‐based eligibility criteria, one of either:
  ○ PLCOm‐2012 risk score [25], inclusion from a threshold of 1.0%–3.2%, evaluated in increments of 0.1% with the addition of 1.51% to mirror the UK TLHC inclusion criteria [10].
  ○ Pack‐year‐based criteria of 10, 20, 30 or 40 pack‐years, paired with a maximum of 10, 15, 20 or 25 years since smoking cessation.

We used a reduced form of the PLCOm‐2012 [25] model, which assumes reference values for socioeconomic covariates as specified in the supplement, and includes the smoking duration, intensity and status of the individual for determining the risk score. As a base case, we assumed perfect adherence to the screening regimen. This allowed us to estimate the expected benefits and harms of screening for the individual screening participant and thus prevents overprescription of screening to combat effects of imperfect attendance [26]. We included a sensitivity analysis that reduced adherence to 50%, split equally between imperfect uptake and imperfect repeat attendance.

### 2.3 Health Economic Inputs

Table 1 shows the health‐economic inputs applied to the events and life years generated by the MISCAN‐Lung model. Costs of lung cancer treatment were applied per person year spent in each of three phases of care: initial (6 months from diagnosis), continuing (intermittent period), and terminal (final 6 months of life), where separate rates were used for those who die of lung cancer and those who die with lung cancer, but of other causes. The complete set of treatment cost rates per stage of cancer, histology, and phase of treatment is reported in Table S1. Treatment costs were estimated by comparing the health care expenditures of the entire Dutch lung cancer patients to their pre‐incidence expenditure levels. Methods and results of this costing analysis were published previously [19].

**TABLE 1:** Cost and quality of life model inputs.

Type of cost/utility	Base value	Description	Index year
Costs in EUR			
Administrative overhead per participant	7.96	Subsidy per participant (16.74) [27] in 2022 for the colorectal cancer screening programme in the Netherlands, less the cost of invitation (0.70) and the Faecal Immunochemical Test (8.08) [28].	2022
Invitation costs	0.70	Invitation cost per screening event, in the 4‐IN‐THE‐LUNG‐RUN trial	2022
Risk assessment	4.60	Cost of sending invitation survey and processing results, in the 4‐IN‐THE‐LUNG‐RUN trial.	2022
Initial LC care phase	2764‐12,004	Treatment costs per month are stratified by the Stage (IA–IV), histology, and phase of care (first 6 months, intermittent, final 6 months), and the period (2013–2017 [little targeted or immunotherapy] and 2018–2021 [more widespread targeted or immunotherapy]). Stratified values are presented in Table S4 of de Nijs et al. [29].	2021
Continuing LC care phase	215–5223		2021
Terminal care—LC death	1816–7164		2021
Terminal care – other cause death	1395–7941		2021
Terminal care in the absence of lung cancer	1048	Average health care costs in the final 6 months of life among those never observed to have lung cancer.	2022
Computed Tomography (CT)	188	CT cost per screening event and follow‐up of false positive screen, using reference cost per National Health Care Institute.	2022
Possibly suspicious screening result	188	Additional CT scan.	2022
Suspicious screening result	660	Dutch Health Care Authority Unit cost for further investigation of a (suspected) malignancy in the respiratory organs. Lump sum for diagnostic imaging, physician renumeration and other hospital expenditures.	2022
Health‐related quality of life			
Terminal LC	0.59	Final 6 months of LC.	—
Stage IA–II LC	0.78	Any life‐year before terminal LC when diagnosed at Stage IA–II.	—
Stage IIIA–IV LC	0.69	Any life‐year before terminal LC when diagnosed at Stage IIIA–IV.	—
Male reference utility	0.864–0.968	The EQ‐5D derived reference utility values for the Netherlands from the EuroQoL group [30]. Values are given by 10‐year age category and sex.	—
Female reference utility	0.746–0.910		—

*Note:* Table reports the health economic parameters applied to MISCAN model outputs to generate QALY gained and incremental costs of screening scenarios relative to no screening. Costs are applied for administration and invitation, for each screening event, for months spent in lung cancer care, and for follow‐up of false positive results. Health‐related quality of life is applied to all life years at a utility specific to the 10‐year age group and sex, obtained from EuroQoL results. For those with lung cancer, a proportional disutility is assigned using the ratio of the lung cancer utilities to the assumed reference group of 65‐year‐olds [31].

Unit costs for screening and follow‐up examinations were derived from the Dutch health care institute costing manual [32]. Invitation and risk assessment costs were taken from the realised values in the 4‐IN‐THE‐LUNG‐RUN screening implementation trial [33]. Administrative overhead was expected to be equal to the subsidy afforded to colorectal cancer screening in the Netherlands, after subtracting the cost of the faecal immunochemical test [27].

To calculate QALYs gained, we assumed Quality of Life under lung cancer care obtained from a systematic review of stage‐ and phase‐specific quality of life among lung cancer patients [34]. Population reference values were taken from the EuroQoL EQ‐5D value set of age‐ and sex‐specific quality of life in the Netherlands [30].

### 2.4 Effectiveness of Smoking Cessation Support

The would‐be eligible screening population is expected to be split equally between former and current smokers [21]. It is considered a best practice to include smoking cessation support as part of the screening programme. As an additional analysis, we simulated the effectiveness of screening assuming the inclusion of a smoking cessation modality. The odds‐ratio of smoking cessation effectiveness is applied to the background cessation rate specific to the age, sex and birth cohort of the individual, per a systematic review of possible cessation modalities applied in the screening context [35]. We assumed pharmacotherapy is offered at each screening event attended by a current smoker, with an OR of cessation of 1.53 (95% CI: 1.33–1.77) [35], and cost of €231.91 per Dutch reimbursement rates [36]. This intervention is the most effective single‐modality reported in the literature, although we previously showed the incremental effects of other smoking cessation interventions [21, 35].

## 3 Results

Cost and benefits were estimated for 1080 strategies of screening implementation. We first present which strategies would be the most efficient (cost‐effective) for implementation in the Netherlands, with particular attention to the required CT capacity for each strategy. We then consider the additional benefits and cost‐effectiveness implications of adding smoking cessation interventions to the simulated CT screening interventions. Finally, we show the sensitivity of our results to model parameters, such as the uptake rate and the cost‐ and health utility inputs.

### 3.1 Cost‐Effective Screening Strategies

Summary outcomes of the most efficient strategies are presented in Table 2. In a no screening scenario, we expect a burden of lung cancer of 5677 incident lung cancers (76.3% Stage III–IV) per 100,000 individuals from the 1945–1979 cohorts over their lifetime.

**TABLE 2:** Summary outcomes of most efficient strategies of lung cancer screening (and 3 reference strategies).

Strat.^a^	Strategy parameters							Cost‐effectiveness					Cancer epidemiology				
	Int.^b^	Start	End	PY	Cess	Risk	Eligible	QALY^c^/100 k	LY^d^/100 k	Cost/100 k	CT^f^/100 k	ICER^e^	LC mort. Red.^g^	Stage III‐IV cancers (/100 k)		Stage I‐II cancers (/100 k)	Frac. overdiag.
NS										€0.00 m			0.0%	4334		1343	
PY1	1	55	75	40	10		8.7%	2605	3301	€28.15 m	106.4 k	€10,805	8.6%	3919	−9.6%	1825	7.7%
PY2	1	50	75	40	10		8.7%	2652	3357	€28.85 m	110.4 k	€14,873	8.6%	3916	−9.6%	1828	7.6%
PY3	1	55	75	30	10		11.5%	3077	3888	€36.16 m	149.6 k	€17,185	10.0%	3847	−11.2%	1906	7.5%
RISK1	1	50	75			1.51%	16.0%	3261	4127	€39.77 m	165.0 k	€19,713	10.8%	3809	−12.1%	1953	7.7%
RISK2	1	50	75			1.3%	17.4%	3402	4301	€42.92 m	184.4 k	€22,235	11.2%	3792	−12.5%	1973	7.7%
RISK3	1	50	75			1.2%	18.2%	3472	4385	€44.66 m	195.3 k	€25,105	11.3%	3783	−12.7%	1983	7.7%
PY4	1	55	80	20	10		14.9%	4006	5104	€60.63 m	248.5 k	€ 29,912	14.9%	3607	−16.8%	2211	9.0%
RISK4	1	50	80			1.2%	23.6%	4325	5524	€70.75 m	290.0 k	€31,638	16.4%	3531	−18.5%	2311	9.4%
RISK5	1	50	80			1.1%	24.7%	4403	5619	€73.42 m	307.3 k	€34,582	16.6%	3519	−18.8%	2326	9.4%
RISK6	1	50	80			1.0%	26.0%	4490	5726	€76.63 m	327.1 k	€36,661	16.9%	3508	−19.1%	2339	9.4%
PY5	1	50	85	20	25		21.9%	4947	6328	€99.03 m	445.5 k	€48,991	20.2%	3333	−23.1%	2582	10.7%
PY6	1	50	85	10	25		28.7%	5350	6830	€124.92 m	621.0 k	€64,286	22.1%	3244	−25.2%	2693	10.7%
TLHC‐A	1	55	75			1.51%	16.0%	3253	4118	€39.69 m	164.6 k		10.8%	3809	−12.1%	1953	7.7%
TLHC‐B	2	55	75			1.51%	16.0%	2276	2912	€29.11 m	94.4 k		7.7%	3967	−8.5%	1781	8.3%
USPSTF	1	50	80	20	15		17.8%	4321	5494	€72.16 m	317.6 k		15.8%	3558	−17.9%	2269	8.9%

*Note:* Reports summary outcomes of the strategies on the efficient frontier in terms of (discounted) cost per Quality‐Adjusted‐Life‐Year gained. Strategy characteristics in terms of starting and stopping age and eligibility criteria (either based on packyears or PLCOm risk) are reported, as well as discounted (3%) costs, LY and QALY gained.

^a^ Strategy shorthand.

^b^ Screening interval.

^c^ Quality Adjusted Life Years.

^d^ Life Years.

^e^ Incremental Cost‐Effectiveness Ratio (extra cost incurred relative to the preceding strategy, divided by the extra QALYs gained).

^f^ Computed Tomography.

^g^ Lung cancer mortality reduction. For reference, but not as part of the efficient frontier, the strategies are reported that are implemented in the UK Targeted Lung Health Check (TLHC‐B – biennial screening ages 55–75 from > 1.51% PLCOm risk, as well as the annual equivalent TLHC‐A), and in the US (USPSTF—annual screening ages 50–80 from 20PY and maximally 15y since cessation).

The efficient screening strategies would include between 8.7% and 28.7% of the population, to reduce total LC mortality by 8.6 to 22.1%. We find the cost‐effectiveness of screening in €/QALY gained to be broadly within common willingness‐to‐pay thresholds, such as the WHO‐recommended 1–3 times GDP (€57.8–173.4 thousand for the Netherlands) [37]. The efficient strategies include both pack‐year‐based and risk‐based criteria. As visualised in Figure S1, biennial or triennial screening strategies are not among the efficient strategies and are dominated by annual screening strategies across the cost‐efficient frontier. This indicates that frequent screening among a high‐risk group is more efficient than infrequent screening among a moderate‐risk group. Overdiagnosis is estimated to be 7.7% (excess incidence) for scenarios screening up to age 75, with higher rates of 9.0%–9.4% expected if screening were to expand up to age 80.

### 3.2 US and UK Screening Strategies

We do not find the strategies of the USPSTF (annual screening 50–80 from 20PY [pack‐years]) or the UK NHS TLHC (biennial screening 55–75 from 1.51% PLCOm risk) to be on the efficient frontier. However, the strategy RISK1 in Table S2, which is cost‐effective with a €17,864/QALY Incremental Cost‐Effectiveness Ratio (ICER) is very close to an annual version of the UK NHS strategy (TLHC‐A), varying only in the starting age of 50 relative to 55 for TLHC‐A. Since only a small portion of screening‐eligibles will cross the 1.51% risk threshold before age 55, strategy RISK1 is very close to TLHC‐A, with RISK1 only producing 14 QALYs per 100,000 individuals over TLHC‐A. This makes TLHC‐A preferred, as it would not require the identification of spurious high‐risk individuals aged 50–55, a potentially resource‐intensive effort.

### 3.3 Capacity Management

Implementation considerations may include capacity management and budget impact. To this end, we include a metric of expected CT volume increase by taking the average CT screening requirement across the first 3 years of implementation and applying a 50% expected uptake rate. This is divided by the current national CT volume per Eurostat, of 2.33 million CT screens [38], to report the expected increase. Figure 1 shows the required increase would be 16.1% for the TLHC‐A strategy (annual screening 55–75 from > 1.51% PLCOm risk). By expanding screening coverage to 1.2% PLCOm risk this would increase to 19.5%. The most significant increase would be posed by expanding screening to age 80% and 1.2% risk. USPSTF2021 would require a 27.5% increase of national CT volume, over five times the average annual growth in CT volume in recent years.

**FIGURE 1:**
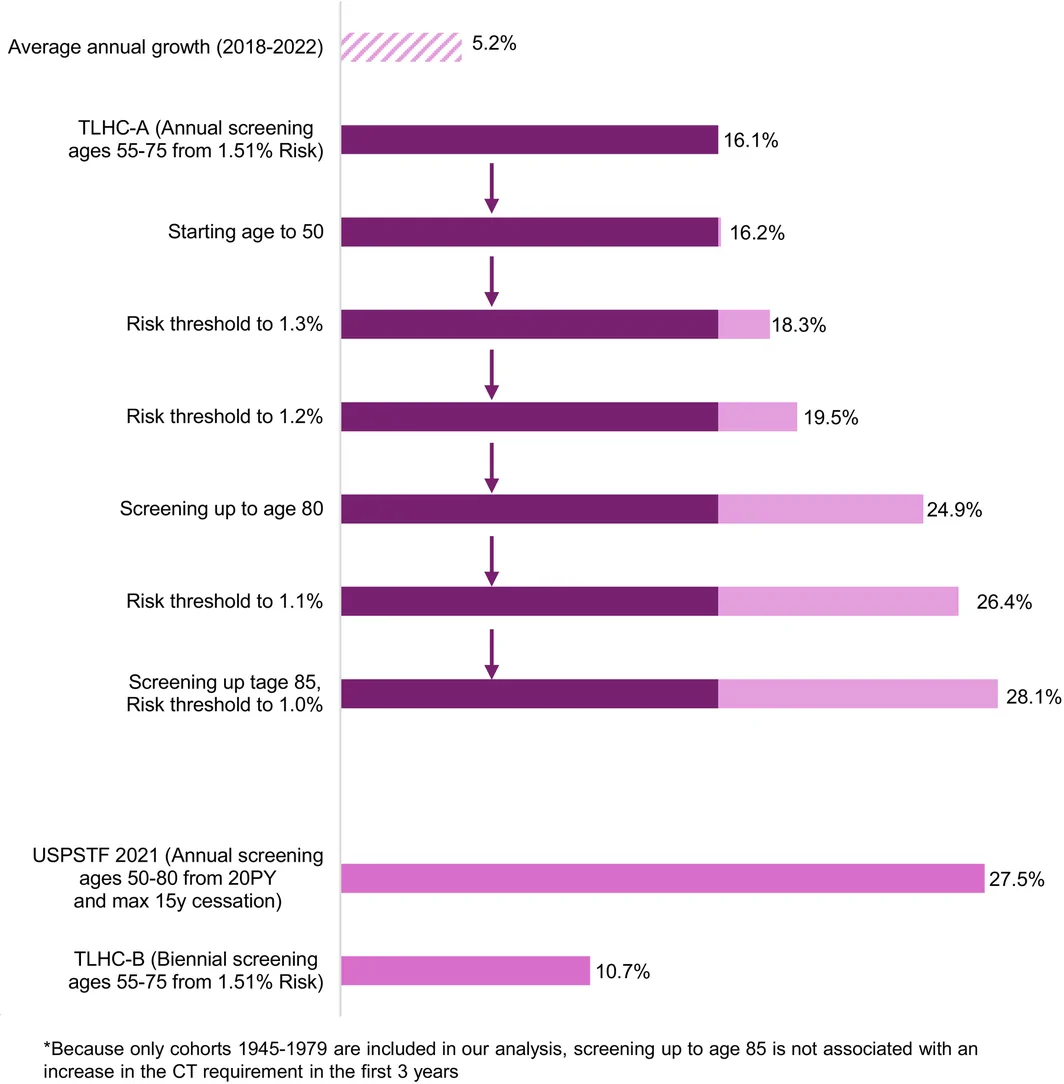
Required CT Capacity increase of strategies to screen Dutch 1945–1979 cohorts, assuming 50% uptake by eligible individuals. This figure shows the CT capacity requirements of the risk‐based strategies on the efficient frontier (as shown in Table 2). The estimated required capacity increase is calculated by the mean number of CT screens in the first 3 years of implementation, multiplied by an assumed uptake rate of 50%. The baseline annual CT screening volume is assumed to be 2.33 million as reported by Eurostat [38]. As also reported in this figure, from 2018 to 2022 the registered CT volume in the Netherlands has grown from 1.81 to 2.33 million, an annualised growth rate of 5.2%.

### 3.4 Budget Impact of Screening

Lung cancer screening implementation will also require allocating financial resources. Figure 2 shows the expected budget impact (the undiscounted cost) by major cost category for the TLHC‐A strategy, assuming a 50% uptake of screening by the eligible population. The primary cost drivers will be CT cost (€1180 million up to 2050) and continuing care for lung cancer (€685 million). Cost savings will be offered by reduced terminal care for lung cancer (€610 million saved). The total expected budget impact will be €2.05 billion, peaking at €283 million in the second year. If we discount future costs by 3%, the net present cost is €1.78 billion.

**FIGURE 2:**
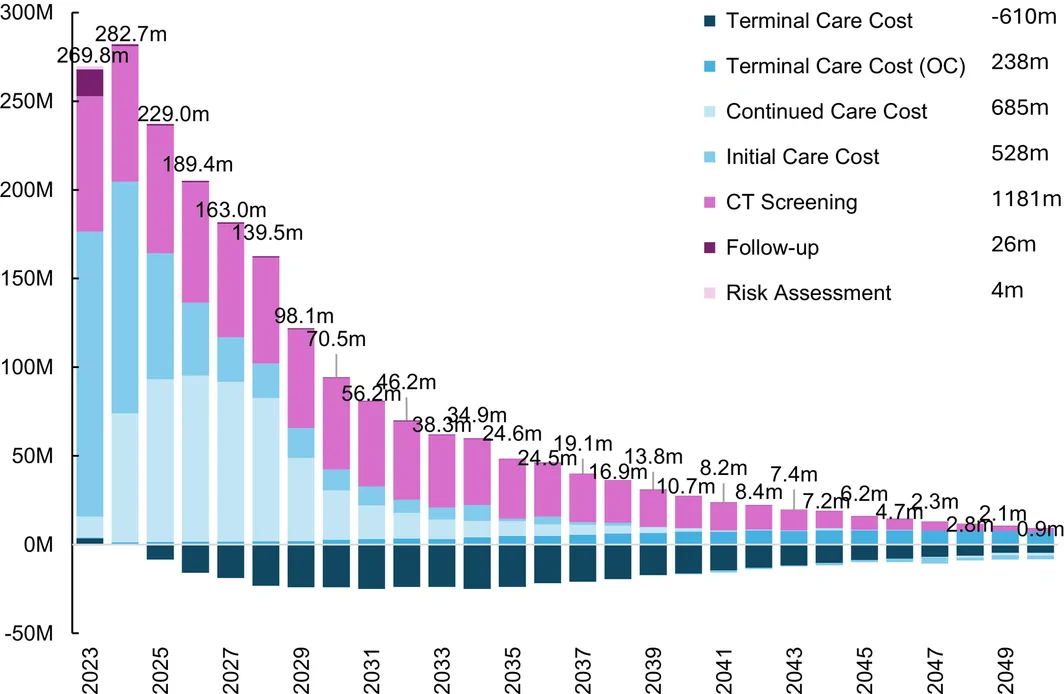
Expected budget impact of lung cancer screening implementation, assuming 50% uptake by eligible individuals. This figure shows the expected budget impact of lung cancer screening implementation (Strategy TLHC‐A per Table 2) for cohorts 1949–1979 in the Netherlands, up to 2050 and scaled to the 2023 population composition. Costs are categorized by treatment cost in phases of care, and the primary cost categories of screening: CT screening, follow‐up exams and risk assessment costs. Invitation and overhead costs are included in the CT screening category. To reflect the per‐year budget impact, costs are not discounted. A 50% attendance rate is assumed, with non‐attendance split equally between structural non‐attenders and incidental non‐attendance.

### 3.5 Additional Smoking Cessation Interventions

Figure 3 shows the shift in QALYs gained and cost of screening if additional smoking cessation interventions are paired with CT screening for current smokers. Both cost and QALY are increased for each strategy on the frontier. Strategies with cessation are generally more cost‐efficient than those without. For the TLHC‐A strategy (per Table S2) we estimate QALY gained of 4282 with smoking cessation, a 32% increase. The addition of smoking cessation to the TLHC‐A strategy has an ICER of €10,041/QALY, making the addition as cost‐efficient as lung cancer screening itself. Our analysis assumes imperfect efficacy, but full uptake of the cessation intervention.

**FIGURE 3:**
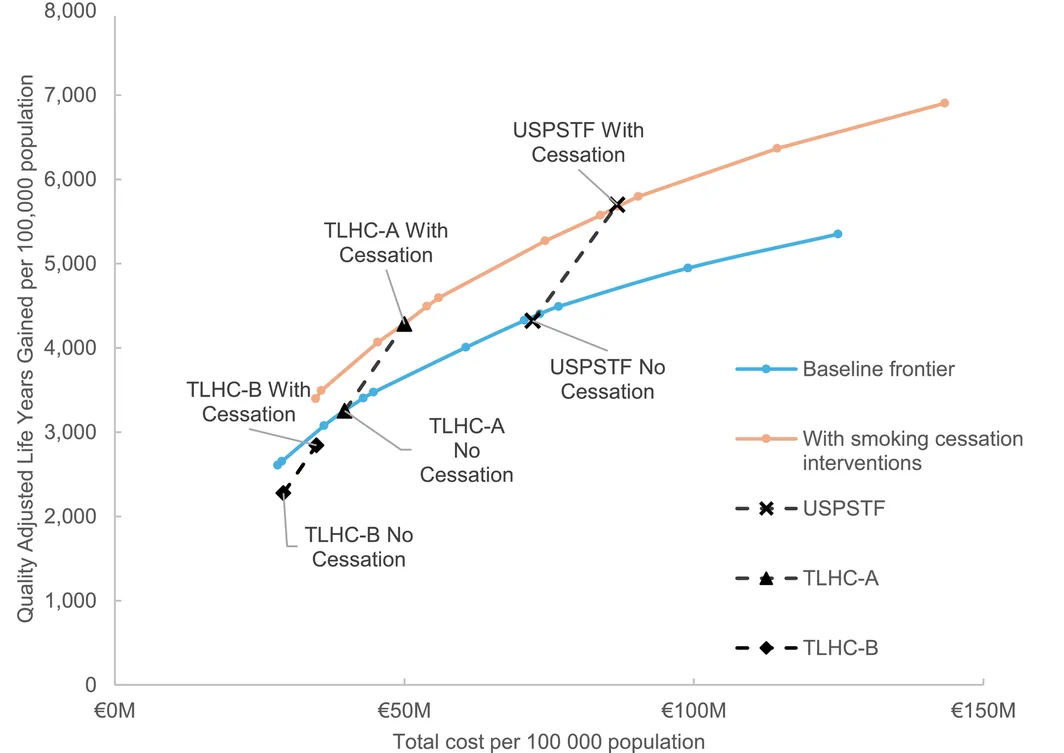
Quality Adjusted Life Years gained and implementation cost per 100,000 population of the most efficient lung cancer screening strategies, with and without supplemental smoking cessation interventions. This figure shows the cost and QALY gained per 100,000 population for strategies on the efficient frontier (presented in Table 2), and the Targeted Lung Health Check (TLHC‐B), the annual equivalent (TLHC‐A) and United States Preventive Services Task Force (USPSTF) 2021 strategies, before and after the addition of smoking cessation interventions. Smoking cessation is assumed to be pharmacotherapy, inducing an odds ratio of 1.53 of smoking cessation to any current smoker attending a CT screening.

### 3.6 Sensitivity of Model Results to Input Parameters

Several sensitivity analyses were performed to test the cost‐effectiveness of lung cancer screening for the Netherlands. Figure S2 shows a univariate sensitivity analysis to major model inputs for the cost‐effectiveness of TLHC‐A and USPSTF strategies relative to no screening. We find the cost‐effectiveness particularly affected by the discount rate employed (TLHC‐A ACER €7732–18,592/QALY for discount rates 0%–6%) and the cost of early‐stage lung cancer treatment (ACER €9042–15,362/QALY for −30 to +30% cost) and CT (ACER €10,049–14,355/QALY for −30 to +30% cost). Attendance and further increases in terminal‐phase care for Stage I and II lung cancer are not found to have strong effects on overall cost‐effectiveness (< 3%). In each scenario, the cost‐effectiveness relative to no screening of TLHC‐A remains below €20,000/QALY. A probabilistic sensitivity analysis of cost and QALY inputs was also performed, shown in Figure S3 for TLHC‐A and USPSTF strategies. The TLHC‐A cost‐effectiveness was within €20,000/QALY for each of 10,000 random draws.

The benefit of a strategy decision may be quantified by the net monetary benefit (NMB) at a given willingness‐to‐pay (WTP) per QALY. By multiplying QALY gained with the WTP, we get the equivalent monetary value of life years gained. Substracting the cost of screening, the NMB is obtained. A suboptimal strategy relative to the efficient frontier generates a loss in NMB, since a strategy with higher NMB would have been possible. Figure S4 shows this expected loss in NMB for a few key strategies, averaged across the 10,000 PSA draws. We find that at a €20 k/QALY WTP, expected NMB is nearly maximised by THLC‐A. At this WTP, the loss in monetary benefit of not implementing screening is 2.0 billion.

## 4 Discussion

Here, we performed an evaluation of the expected benefit and health‐economic outcomes of 1180 possible targeted strategies of lung cancer screening with CT for the Dutch population. We considered the cost‐effectiveness in cost per QALY gained, as well as the required CT volume for implementation and the effects of adding smoking cessation interventions. We found both pack‐year‐based and PLCOm risk‐based criteria to offer cost‐efficient avenues of risk selection. At a €20 k/QALY WTP threshold, the optimal strategy would either be the annual equivalent of the UK Targeted Lung Health Check strategy of screening ages 55–75 from a 1.51% PLCOm risk (although we suggest to forego the 0.01% for practicality) or further including all those with 1.51% risk from age 50 (although this includes a negligible additional share of the population). The TLHC‐A strategy would reduce lung cancer mortality by 10.8% at full uptake, and 6.2% at 50% uptake, equivalent to 1072 and 615 LC deaths prevented annually relative to 2023 lung cancer deaths in the Netherlands.

If half of eligible individuals attend screening, the national CT volume would need to increase by 16.1% to accommodate a screening programme. Extending screening further to those with a lower risk, or up to age 80, would further reduce the mortality burden of lung cancer. In cost per QALY gained, these would also still be cost‐effective by many measures, with screening 50–80 from 1.0% PLCOm costing €36 k per QALY gained. However, this would require a 28% increase in CT volume [39]. In all, we recommend implementation of an annual strategy, to screen ages 55–75 from 1.5% risk, which is near‐optimal and offers a feasible trajectory for scaling up lung cancer screening. From early experience of CT screening implementation from the 4‐IN‐THE‐LUNG‐RUN trial, screening CTs are feasible in less than half the time of diagnostic CT thorax (5–7 min as opposed to 15–20). The 16.1% required CT volume increase for TLHC‐A may therefore correspond to an 8% increase in CT‐hours, which could ease capacity management. Implementation of lung screening would likely be spread over a number of years, as demonstrated by the UK program, and the implementation of other screening programmes in the Netherlands [40, 41]. More mature screening programmes may consider expansion to lower thresholds of eligibility or higher stopping ages but should consider further capacity requirements.

We find smoking cessation programmes to cost‐effectively yield 30% extra QALY gained from screening. This includes both additional benefits in lung cancer prevention, as well as benefits to other causes of smoking‐related mortality. This is higher than our previous model‐based estimates of 20% [21], although those only considered a 60–80 population with a very high risk of developing lung cancer, for whom additional smoking cessation offers lower lifetime benefit. Our analysis pairs cessation interventions with screening events, and therefore only offers a rudimentary view of the potential integration of smoking cessation and lung cancer screening programmes. In practice, smoking cessation services could be offered to a broader population and provide an avenue for identifying those ever‐smokers for whom lung cancer screening is warranted, while also personalising smoking cessation modalities to better suit individual needs. Given the benefits to reducing other non‐lung cancer causes of morbidity and mortality, smoking cessation interventions may be distributed to younger and lower‐risk individuals than the cost‐effective lung cancer screening population. This may offer earlier, more effective smoking cessation, and potentially prevent individuals from ever needing to enter screening. Lung cancer screening frequency and coverage must balance the benefits against harms such as overdiagnosis and false positives, where smoking cessation does not. This makes it likely that the optimal smoking cessation intervention does not restrict itself only to the screening‐eligible population. We also considered only one example smoking cessation modality (pharmacotherapy) using reported effectiveness from a meta‐analysis. More data from real‐world screening programmes may inform whether more efficacious or cost‐efficient methods are feasible [35, 42, 43].

Similar to previous estimates from both the MISCAN‐Lung model and other health‐economic evaluations, we find CT screening to be increasingly cost‐efficient [13, 14, 15, 16]. Considering increasing late‐stage lung cancer treatment costs and after recalibration of the model to NELSON evidence, we find an ICER of TLHC‐like screening of only €12,202/QALY (relative to no screening). For the countries of Switzerland and Australia, this was €19,341/QALY and AUD62,754, respectively, before integrating NELSON evidence [13, 15]. We find the cost‐effectiveness to be particularly sensitive to the cost of CT screening. In practice, the cost of screening may be further reduced by economies of scale, integration of artificial intelligence, and personalised screening intervals. The ongoing 4‐IN‐THE‐LUNG‐RUN trial, across five European countries, is studying the non‐inferiority of biennial screening for those with a negative baseline screen, after NELSON results showed baseline results to be highly indicative of future lung cancer risk [33, 44, 45]. Future research may consider the implications of such personalised follow‐up, which may offer an avenue towards lower resource use or restrained screening for a larger targetable group. Additionally, the ICERs may currently underestimate the cost‐effectiveness of screening if novel treatments have further increased the cost of late‐stage treatment, as demonstrated by the univariate sensitivity analysis in Figure S2.

After incorporating NELSON evidence, the evolving lung cancer treatment landscape, and the potential for integrated smoking cessation support, we consider lung cancer screening to be confidently cost‐efficient. A risk‐based strategy screening from 1.5% PLCOm risk between ages 55–75 can be implemented within feasible growth of CT volume.

## Author Contributions

**Koen de Nijs:** conceptualization, writing – review and editing, methodology, investigation, visualization, validation, writing – original draft. **Kevin ten Haaf:** writing – review and editing, conceptualization, supervision, project administration, funding acquisition, methodology. **Dana Moldovanu:** conceptualization, writing – review and editing. **Juul Hubert:** conceptualization, writing – review and editing. **Isabelle van den Bosch:** conceptualization, writing – review and editing. **Carlijn van der Aalst:** conceptualization, writing – review and editing, funding acquisition, project administration. **Harry J. de Koning:** conceptualization, writing – review and editing, funding acquisition, project administration, supervision, methodology.

## Funding

This work was supported by HORIZON EUROPE Health, 848294; Nederlandse Organisatie voor Wetenschappelijk Onderzoek, 09150161910060.

## Ethics Statement

As the study constitutes a cost‐effectiveness modelling study, no patient‐level information was processed, and no ethical review or informed consent was required.

## Conflicts of Interest

Koen de Nijs reports grants to his institution from the NIH (U01CA253858), the European Commission (848294) and the University of Zurich. Dr. ten Haaf reports grants from Dutch Research Council/Netherlands Organisation of Health Research (ZonMW Grant number 09150161910060) and European Union (Horizon 2020, 848,294.) during the conduct of the study, external to the study he reports grants from NIH/National Cancer Institute Grant (1U01CA199284–01) grants from University of Zurich, Switzerland, grants from Cancer Research UK, grants from Cancer Australia, grants from Medical Services Advisory Committee of the Australian Ministry of Health, grants from Open Mind Call Convergence, speaking and consulting fees, and travelling reimbursements from Centre hospitalier universitaire vaudois, Johnson&Johnson, Deutsches Krebsforschungszentrum, SkinVision, paid to their institute. Travelling reimbursements from Rescue Lung Society and International Association for the Study of Lung Cancer and personal prize from Heineken Prizes Foundations. Carlijn van der Aalst reports grants from the European Commission (payment to her institution): 4‐IN‐THE‐LUNG‐RUN (848294), TSI‐2025‐LLICS‐IA (101262491), PREVALUNG EU (101095604), TSI‐LUKA‐Slovenia, and IARC‐WHO: European Code Against Cancer. Harry de Koning reports grants from the NIH, speaker fees from AstraZeneca and advisory board membership to the SOLACE consortium. The other authors declare no conflicts of interest.

## Supporting information


**Table S1:** Summary outcomes of most efficient strategies of lung cancer screening at 50% attendance of screening events.
**Table S2:** Summary outcomes of most efficient strategies of lung cancer screening with concomitant smoking cessation support.
**Figure S1:** Discounted cost and Quality‐Adjusted Life Years gained of screening strategies by starting age and screening interval.
**Figure S2:** Univariate sensitivity analysis of key model inputs.
**Figure S3:** Probabilistic Sensitivity Analysis of Cost and QALY inputs for the cost‐effectiveness of TLHC‐A and USPSTF 2021 strategies.
**Figure S4:** Expected loss in net monetary benefit relative to efficient frontier by screening strategy and Willingness‐to‐Pay per Quality‐Adjusted Life Year.
**Figure S5:** Other‐Cause Mortality Probability by Age and Smoking Status for the Male 1960 cohort.
**Figure S6:** Lung cancer progression in the MISCAN‐Lung model.
**Figure S7:** Integrating modules: modelling benefits of screening.
**Figure S8:** Integrating modules: modelling harms of screening.
**Figure S9:** MISCAN‐Smoking History Generator (SHG) predictions of current smoking prevalence compared to observed Dutch Health Survey smoking prevalence for Male 10‐year cohorts 1930–1970.
**Figure S10:** MISCAN‐Smoking History Generator (SHG) predictions of current smoking prevalence compared to observed Dutch Health Survey smoking prevalence for Female 10‐year cohorts 1930–1970.
**Figure S11:** MISCAN‐Lung simulated lung cancers by age and sex for the period 2000–2020 in the Netherlands.

## Data Availability

MISCAN model outputs and further data that support the findings of this study are available from the corresponding author upon reasonable request.
