# Supplementary material for: The Optimal Lung Cancer Ct Screening Strategy: A Cost‐Effectiveness Study Incorporating Nelson Evidence, Evolving Cost of Treatment and Smoking Cessation Interventions
Source: Int J Cancer. 2026 Jul 29;159(12):2886–96. doi: 10.1002/ijc.70655 (PMC13688233; doi:10.1002/ijc.70655)
Supplement: Supplementary file 1 [file IJC-159-2886-s001.pdf]

Supplementary Materials supporting *"The optimal lung cancer CT screening strategy: A cost-effectiveness study incorporating NELSON evidence, evolving cost of treatment and smoking cessation interventions."*

Koen de Nijs, Kevin ten Haaf, Dana Moldovanu, Juul Hubert, Isabelle van den Bosch, Carlijn van der Aalst, Harry J. de Koning

TABLE OF CONTENTS

Supplementary Materials supporting *"The optimal lung cancer CT screening strategy: A cost-effectiveness study incorporating NELSON evidence, evolving cost of treatment and smoking cessation interventions."* 1

Methodological Supplement 2

- Model summary and structure 2
- Smoking history generator 3
- Death from other causes 3
- Smoking dose-response relationship for lung carcinogenesis 3
- Natural History of Lung Cancer 4
- Computed Tomography Screening 4
- Integrating Modules 4
- Smoking Cessation Interventions 7
- Model Outcomes 7
  - Adaptation of NELSON results to expected false positive rates 8
- Dutch smoking behavior and lung cancer epidemiology 10
- NELSON Screening Effectiveness 11
- References 11

Supplementary Tables 13

Supplementary Figures 17

Methodological Supplement

Model summary and structure

To estimate the effectiveness of lung cancer screening, as well as additional smoking cessation support, we used the MIcrosimulation SCreening ANalysis (MISCAN) Lung model, a stochastic, microsimulation model. In brief, the model simulates individual life histories in the considered population from birth until death, in the presence or absence of a screening program. Through comparing the life histories in the presence of screening with the corresponding life histories in the absence of screening, MISCAN-Lung can estimate the effectiveness and costs of screening scenarios. MISCAN-Lung was calibrated to individual-level data from the National Lung Screening Trial (NLST) and the Prostate, Lung, Colorectal, and Ovarian Cancer Screening Trial (PLCO),[1 2] and has since been recalibrated to the Nederlands-Leuven Longkanker Screening Onderzoek (NELSON).[3] Estimates of the preclinical sojourn time by stage, and the probability of lung cancer detection are now matched to outcomes from the NELSON trial, per methods elaborated on in a future section. The model is programmed in Python 3.

MISCAN-Lung is a semi-Markov model, which generates durations for each state. Individuals are simulated one at a time, which allows future state transitions to depend on past transitions giving the model a “memory”. MISCAN-Lung simulates sequences of events by drawing from distributions of probabilities/durations, which makes the results of the model subject to random variation. The model is calibrated such that the aggregate of all these individual outcomes matches the smoking behavior patterns and lung cancer incidence across different sexes and birth cohorts of the Netherlands, and the outcomes of the PLCO, NLST and NELSON trials. For smoking behavior prevalences, we use the Dutch Health Survey for years 1989 to 2020 to establish proportions of current, former and never smokers over time for each 5-year birth cohort and sex separately.[4] Lung cancer incidence is taken from the Netherlands Cancer Registry, covering all incident lung cancers 2000-2020 in the Netherlands by stage and histology of cancer.[5]

MISCAN-Lung consists of several modules: a demography/smoking history generator module, a smoking-dose response module for lung carcinogenesis, a natural history module and a screening module. The workings of each of these modules will be discussed in detail in the coming sections.

Smoking history generator

First, birth-tables, representative for the population under consideration, are used to draw a date of birth for each simulated individual. Age, sex and five-year birth-cohort specific smoking initiation probabilities, representative for the population under consideration, are used to determine whether an individual initiates smoking and the age of smoking initiation. Upon smoking initiation, persons enter one of five increasing smoking intensity categories. Age, sex, five-year birth-cohort and smoking intensity category specific by averaged number of cigarettes smoked per day are generated for each individual that initiates smoking. If an individual initiates smoking, age, sex and cohort specific smoking cessation probabilities are used to determine whether an individual ceases smoking and the age of smoking cessation. The workings of additional smoking cessation interventions, which may cause smoking cessation in excess of the background rate, are discussed in the section `smoking cessation interventions`.

Death from other causes

Upon generating a person's smoking history, the age of death from causes other than lung cancer is generated, using mortality probabilities based on the person's smoking history (smoking duration, smoking intensity category and average number of cigarettes per day, smoking status and years since cessation, if applicable), year of birth, age and sex. For example, for the Male 1960 cohort, Supplementary Figure 5 shows the mortality rate for never-smokers relative to current smokers for each smoking intensity quintile. After smoking cessation, individuals are assigned a mortality probability in between the never- and current-smoker mortality probability, weighted by their years since smoking cessation. The maximum age an individual can achieve in MISCAN-Lung is exactly 100 years.

Smoking dose-response relationship for lung carcinogenesis

The smoking-dose response module allows modeling lung carcinogenesis as a function of a person's age, gender and smoking history. MISCAN-lung utilizes the two-stage clonal expansion model (TSCE) as described by Heidenreich et al., as its smoking-dose response module (which estimates a person's risk of lung cancer, as a function of age and smoking history).[6] The parameters of the TSCE were obtained through calibration to the Nurses' Health Study and the Health Professionals Follow-up Study [7], and were further calibrated to Dutch lung cancer incidence, smoking behavior, and

individual-level outcomes from the NELSON study. [4, 5, 8]

Natural History of Lung Cancer

Lung cancers are assumed to progress sequentially through stages IA to IV, as shown in Supplementary Figure 6. The probability that a lung cancer progresses to a more advanced preclinical stage or is diagnosed clinically (e.g., diagnosed due to symptoms) is modelled by histology and stage. After clinical diagnosis, lung cancer survival is simulated using sex-, stage-, and histology specific survival estimates, obtained from the Netherlands Cancer Registry.[5] The date of death for individuals with lung cancer is set to the earliest simulated date of death (either due to lung cancer or other causes).

Computed Tomography Screening

Screening may detect cancers in each of the preclinical screen-detectable states, depending on the sensitivity of the screening test for the specific histology and preclinical stage. The model parameters for CT sensitivity by preclinical stage and histology and the effectiveness of CT screening were calibrated to individual-level data from the NELSON trial.[3] Upon detection of lung cancer by screening, a person's life history may be altered. Detection by screening may prevent the lung cancer death, allowing them to resume their normal (lung cancer free) life history. The date of death then reverts to the stochastically generated date of death from other causes. The life years gained are the difference between this other-cause death and the lung cancer death – if it would have occurred without the lung cancer screening. The probability of successful mortality prevention differs by the stage at detection, and was also calibrated to individual-level outcomes from the NELSON study – by fitting the rate of death in the screening arm in a model reproduction of the trial to the NELSON outcomes. If the screen detection of the cancer is unsuccessful at preventing lung cancer mortality (the probability of cure fails) – the same date of death from lung cancer is assumed as in the no-screening scenario. Negative effects of screening, such as overdiagnosis of lung cancer (described subsequently), are also modelled.

Integrating Modules

Supplementary Figure 7 shows an example of how the model integrates the different modules to determine the benefits of screening. The demography/smoking history generator module first

generates a date of birth, smoking history and date of death from causes other than lung cancer. This creates a life-history in the absence of lung cancer for Person 1 (shown in life history 1). The smoking-dose response module uses the simulated smoking history to determine whether and when lung carcinogenesis occurs for Person 1 (shown in life history 2). After lung carcinogenesis occurs, the natural history model generates the progression of the cancer, which is diagnosed because of symptoms in stage II in this example and results in a death due to lung cancer, before the death due to causes other than lung cancer would have occurred (shown in life history 1). In the screening module, a screening examination is simulated, as indicated by the arrow (shown in life history 3). The cancer is detected at the examination and, in this case, the earlier detection allows for successful treatment of the cancer. As a result, the lung cancer death is prevented, and the person's life is prolonged.

Screening may also cause harms, as shown for Patient 2 in Supplementary Figure 8 In Patient 2 lung cancer also develops, but the cancer would not have been clinically detected without screening (shown in life history 2). However, the cancer is screen-detected in stage IA during the screening examination simulated in the screening module (shown in life history 3). Thus, in this patient, screening detects a lung cancer that would have never become apparent during the patients' lifetime if screening had not occurred, resulting in an overdiagnosed case. Thus, for Patient 2 screening does not provide any benefits, but results in life-years with lung cancer care that would not have occurred otherwise (overtreatment).

Smoking Cessation Interventions

In this particular study, we add a model component to facilitate interventions aimed at inducing smoking cessation. An individual may be scheduled to attend a smoking cessation intervention at a given age. At a probability adjusted for the assumed odds-ratio of the intervention, and the background rate of smoking cessation, the individual may permanently quit smoking instantaneously. The number of cigarettes smoked per day for the years from that point forward is set to 0. The other-cause mortality age and age of lung cancer onset (if no preclinical lung cancer is already present at the age of smoking cessation) originally estimated for the individual for a life-history without smoking cessation interventions are re-estimated to incorporate the change in smoking behaviour. The initial random thresholds of cancer onset and other-cause mortality are preserved, such that the new probability of age can only result in a later age of onset or other-cause mortality.

To obtain the instantaneous probability of cessation from the odds ratio, we multiply the background odds of smoking cessation by the odds ratio, convert these odds to the equivalent overall cessation probability, from which we subtract the background cessation rate. For example, if we have a background cessation rate of 5% and an odds ratio of 1.5 for a given intervention, the background cessation odds are 1 to 19. In the intervention group, we expect odds of 1.5 to 19, or 1 to 12.67, equivalent to a total cessation rate of 7.3%. To get to a cessation rate of 7.3% in the intervention group, we apply an instantaneous cessation probability of 2.4% (1 – 0.927/0.950) at the moment of the intervention.

We obtain odds-ratios by smoking cessation support modality from the literature[9]. We use the odds ratios obtained from a meta-analysis for all studies reporting 6-month point prevalence of smoking status. We forego the 12-month estimates, because although a longer follow-up is associated with more definitive smoking cessation, only a subset of studies report smoking cessation at 12 months. To account for the uncertainty surrounding the smoking cessation odds ratios, we evaluate lung cancer outcomes using the point estimate odds ratios, as well as at the edges of the 95% confidence intervals (CIs) of the reported odds ratios. We assume the efficacy of pharmacotherapy (OR 1.53, CI 1.33-1.77), with estimates for previous cessation methodologies published previously.[10]

Model Outcomes

The model outcomes presented in Table 1 of the main manuscript are generated as follows:

- **LC Cases:** The sum of lung cancer incidence events, including screen-detected lung cancers.
- **LC Deaths:** The sum of lung cancer death events.
- **LC Deaths Averted:** The sum of lung cancer death events, less the sum of lung cancer deaths events in the scenario without interventions (screening or smoking cessation support).
- **Overdiagnosed Cancers:** The number of screen-detected lung cancers that would not have been clinically detected in the scenario without screening. This is calculated by the excess incidence, that is the increase in incidence in the screening scenario relative to the scenario without screening. When smoking cessation interventions are included, it is calculated as the excess incidence relative to the scenario with only the smoking cessation intervention and no screening.

- **False positives:** The number of false-positive screening results, per rates adapted from the NELSON trial results.
- **Life-years:** Remaining total life-years lived measured from the start of screening (2022).
- **Life-years gained per death averted:** Life-years divided by the `LC Deaths Averted`.
- **CT Screens:** Total required Computed Tomography screens.
- **Screens per life-year gained:** `CT screens` divided by `Life-years`.
- **Proportion eligible:** The share of the given population who are eligible for at least one round of CT screening from 2022 onwards.

Adaptation of NELSON results to expected false positive rates

To represent lung cancer screening protocols as expected to be implemented in the Netherlands, we assume nodule follow-up to conform to the protocol of the ongoing 4-IN-THE-LUNG-RUN trial. To estimate the rate at which each 4-IN-RADS category is registered, we use nodule size and growth distributions adapted from published NELSON results. The 4IN rads categories define separate size categories for each of 4 possible categorisations. We calculate the probability of a false positive, as being the probability of incurring the given nodule category inducing follow-up exams (3 to 4), without having a subsequent lung cancer diagnosis. This probability is derived by applying the rate of cancer diagnosis for each nodule category, as well as the overall probability of having no cancer, to Bayes' theorem. The resulting false positive rates (underlined in the table below) are used to calculate the resource requirement of managing false positive screening results. For nodule category 3, this constitutes a repeat CT scan. For nodule category 4, this is expected to constitute a diagnostic follow-up trajectory, as priced by Dutch health insurers. For subsequent screening, the total false positive rate is the sum of the probability of incurring a category 3 or 4 classification for existing as well as new nodules.

The distribution of nodule volume at baseline, as well as the distribution of volume doubling time in subsequent rounds was derived from Table 1 of Horeweg et al. (2014).[11]

Nodule growth rate and volume of new nodules was taken from Table 3 of Walter et al. (2016).[12]

	Baseline			Subsequent screening					
				Existing nodules			New nodules		
4-IN-THE-LUNG-RUN Category	Overall prob. (%)*	Cancer diagnosis prob.(%)	Prob. if no cancer (%)**	Overall prob. (%)*	Cancer diagnosis prob.(%)	Prob. if no cancer (%)	Overall prob. (%)*	Cancer diagnosis prob. (%)	Prob. if no cancer (%)
**Negative (1)**<br>No nodules or calcified	55.3	0.4	55.9	79.5	0.8	79.9			
**Benign (2)****<br><100 mm^3 at baseline<br>Or VDT > 600 days<br>or new nodule <50mm^3	32.2	0.7	32.5	10.2	0.4	10.2	2.9	0.5	3.0
**Possibly Suspicious (3)****<br>100-300mm^3 at baseline<br>or VDT 400-600 days<br>or new nodule 50-200mm^3	7.8	2.7	7.7	1.2	4.9	1.2	3.8	3.1	3.7
**Suspicious (4)**<br>>300mm^3 at baseline<br>or VDT <400 days<br>or new nodule >200mm^3	4.7	19.2	3.8	1.2	15.4	1.0	1.2	16.9	1.0

* The probability of the nodule category occurring in this round, with the probability of nodule categories across new and existing nodules summing to 100% in subsequent screening.

**Derived by calculating the probability of the given category, conditional on not being subsequently diagnosed with lung cancer. i.e. $P(Cat|NC) = P(NC|Cat) * P(Cat)/P(NC)$. For example, for category II at baseline, in NELSON 32.2% presented with a nodule <100 mm^3. The overall cancer rate in this group was 0.7%, and 1.53% in all baseline participants (1.26% in all subsequent screening). This gives $P(2|NC) = \frac{(1-0.007)*0.322}{1-0.0153} = 32.5\%$.

***Note that some thresholds were simplified to conform to reported NELSON size bins, particularly those new nodules 30-50mm^3 which are category 3 in the 4-IN-THE-LUNG-RUN, were not reported separately in NELSON reports of nodule volume. We also do not account for subsolid nodules which were not reported on separately, despite having distinct categorization thresholds in the nodule management protocol.

Dutch smoking behavior and lung cancer epidemiology

To calibrate the MISCAN-Lung model inputs to the Dutch setting, we evaluated the model's potential to replicate Dutch lung cancer incidence. This ensures that model is representative of broader lung cancer epidemiology in the Netherlands.

We simulate lung cancer outcomes for the years 2000-2020 for cohorts 1935 to 1979. Smoking initiation, cessation and smoking-related other-cause mortality are calibrated to cohort life tables from the bureau of statistics and smoking prevalence per the Dutch Health Survey (1989-2020).[5, 13] Microdata from the Dutch Health Survey informs our estimates of the quintiles of smoking intensity in cigarettes per day, by sex and 5-year cohort.[4] National tobacco sales contemporary to the Dutch Health Survey are used to evaluate quantity underreporting by health survey respondents, yielding an estimate of 21% underreporting, consistent with previous estimates for other contexts[14-16]. Together, these estimates of smoking behavior are used to inform the Smoking History Generator component of the MISCAN-Lung model. The fit of the smoking history generator to observed current smoking prevalence over time by cohort are given in Figures 9 and 10.

For a given set of model inputs, 10,000,000 life histories are simulated. The sizes of the individual cohorts constituting this population are set to comply to 2010 cohort sizes per Statistics Netherlands. For each simulated individual, a smoking history and smoking related other-cause date of death are drawn. Depending on the smoking history, a lung cancer natural history may be established, using the Two-Stage Clonal Expansion model. Lung cancer outcomes are noted for the years 2000-2020 to compare to population-level calibration targets of lung cancer incidence. Simulated and recorded outcomes include lung cancer incidence and mortality by histology (adenocarcinoma, squamous cell carcinoma, other non-small cell lung cancer, and small cell lung cancer) and stage of cancer at incidence (stages IA, IB, II, IIIA, IIIB and IV). Figure 11 shows the lung cancer incidence generated after model calibration by the MISCAN-Lung model for the period 2000-2020, relative to observed values from the Dutch Cancer Registry.

NELSON Screening Effectiveness

Additionally, the model was calibrated to individual-level outcomes from the NELSON study to re-estimate parameters of screening effectiveness first calibrated to results from the NLST. [1, 3, 8] The model calibration replicates the life history of each NELSON participant, and sets the MISCAN-Lung parameters of screening effectiveness to the values that best match the observed outcomes in the NELSON trial. Self-reported smoking histories were used to replicate lung cancer outcomes among simulated NELSON participant. Recalibrated parameters include the sensitivity of the CT screen by stage, as well as the preclinical sojourn time in stage IA Adenocarcinoma. NELSON calibration results suggest a higher sensitivity of CT screening for the detection of lung cancer than previous results from NSLT and PLCO trials, as well as a longer period of detectability in stage IA Adenocarcinoma. [1, 2] The complete NELSON-adjusted MISCAN-Lung parameter set and calibration methodology are summarized in a separate publication.[8]

References

1. ten Haaf K, van Rosmalen J, de Koning HJ. Lung Cancer Detectability by Test, Histology, Stage, and Gender: Estimates from the NLST and the PLCO Trials. *Cancer Epidemiology Biomarkers & Prevention* 2015;**24**: 154.

2. Meza R, ten Haaf K, Kong CY, Erdogan A, Black WC, Tammemagi MC, Choi SE, Jeon J, Han SS, Munshi V, van Rosmalen J, Pinsky P, et al. Comparative analysis of 5 lung cancer natural history and screening models that reproduce outcomes of the NLST and PLCO trials. *Cancer* 2014;**120**: 1713-24.

3. de Nijs K, ten Haaf K, van der Aalst CM, Oudkerk M, de Koning HJ. OA05.04 A Comparison of Stage- and Histology-Specific CT Sensitivity in the NELSON Trial and the NLST. *Journal of Thoracic Oncology* 2022;**17**: S14.

4. Statistics Netherlands. Dutch Health Survey, 1989-2020.

5. Netherlands Cancer Registry (NCR). Lung Cancer Incidence and Survival. In: Netherlands Comprehensive Cancer Organisation (IKNL), ed., 2000-2012.

6. Heidenreich WF, Luebeck EG, Moolgavkar SH. Some properties of the hazard function of the two-mutation clonal expansion model. *Risk Anal* 1997;**17**: 391-9.

7. Meza R, Hazelton WD, Colditz GA, Moolgavkar SH. Analysis of lung cancer incidence in the nurses' health and the health professionals' follow-up studies using a multistage carcinogenesis model. *Cancer Causes & Control* 2008;**19**: 317-28.

8. de Nijs K, Ten Haaf K, Hubert J, Moldovanu D, van der Aalst CM, Groen HJM, de Jong PA, Heuvelmans MA, Oudkerk M, de Koning HJ. Stage- and histology-specific sensitivity for the detection of lung cancer of the NELSON screening protocol-A modeling study. *Int J Cancer* 2025.

9. Cadham CJ, Jayasekera JC, Advani SM, Fallon SJ, Stephens JL, Braithwaite D, Jeon J, Cao P, Levy DT, Meza R, Taylor KL, Mandelblatt JS, Collaboration C-S. Smoking cessation interventions for potential use in the lung cancer screening setting: A systematic review and meta-analysis. *Lung Cancer* 2019;**135**: 205-16.

10. de Nijs K, Ten Haaf K, van der Aalst C, de Koning HJ. Projected effectiveness of lung cancer screening and concurrent smoking cessation support in the Netherlands. *EClinicalMedicine* 2024;**71**: 102570.

11. Horeweg N, van Rosmalen J, Heuvelmans MA, van der Aalst CM, Vliegenthart R, Scholten ET, ten Haaf K, Nackaerts K, Lammers JW, Weenink C, Groen HJ, van Ooijen P, et al. Lung cancer probability in patients with CT-detected pulmonary nodules: a prespecified analysis of data from the NELSON trial of low-dose CT screening. *Lancet Oncol* 2014;**15**: 1332-41.

12. Walter JE, Heuvelmans MA, de Jong PA, Vliegenthart R, van Ooijen PMA, Peters RB, Ten Haaf K, Yousaf-Khan U, van der Aalst CM, de Bock GH, Mali W, Groen HJM, et al. Occurrence and lung cancer probability of new solid nodules at incidence screening with low-dose CT: analysis of data from the randomised, controlled NELSON trial. *Lancet Oncol* 2016;**17**: 907-16.

13. Statistics Netherlands. Levensverwachting; geslacht, leeftijd (per jaar en periode van vijf jaren), 2020.

14. Pérez-Stable EJ, Marín BV, Marín G, Brody DJ, Benowitz NL. Apparent underreporting of cigarette consumption among Mexican American smokers. *American Journal of Public Health* 1990;**80**: 1057-61.

15. Gallus S, Tramacere I, Boffetta P, Fernandez E, Rossi S, Zuccaro P, Colombo P, La Vecchia C. Temporal changes of under-reporting of cigarette consumption in population-based studies. *Tobacco Control* 2011;**20**: 34-9.

16. Liber AC, Warner KE. Has Underreporting of Cigarette Consumption Changed Over Time? Estimates Derived From US National Health Surveillance Systems Between 1965 and 2015. *Am J Epidemiol* 2018;**187**: 113-9.

Supplementary Tables

Supplementary Table 1– Summary outcomes of most efficient strategies of lung cancer screening at 50% attendance of screening events

Strategy Parameters								Cost-effectiveness					Cancer Epidemiology			
Strat. *	Int.†	Start	End	PY	Cess	Risk	Eligible	QALY‡ /100k	LY§ /100k	Cost /100k	CT# /100k	ACER¶	LC Mort. Red. **	Stage III-IV Cancers (/100k)	Stage I-II Cancers (/100k)	Frac. Overdiag.
NS										€0.00 m			0.0%	4,334	1,343	
PY1	1	55	75	40	10		8.7%	1,469	1,871	€16.45m	55.0k	€11,202	4.9%	4,098	1,621	8.1%
PY2	1	50	75	40	10		8.7%	1,494	1,901	€16.94m	57.0k	€11,336	4.9%	4,097	1,622	8.0%
PY3	1	55	75	30	10		11.5%	1,743	2,212	€20.88m	77.2k	€11,981	5.7%	4,056	1,668	7.9%
RISK1	1	50	75			1.51%	16.0%	1,841	2,341	€22.99m	85.7k	€12,490	6.2%	4,036	1,694	8.1%
RISK2	1	50	75			1.3%	17.4%	1,919	2,437	€24.54m	95.7k	€12,784	6.4%	4,026	1,706	8.1%
RISK3	1	50	75			1.2%	18.2%	1,957	2,484	€25.56m	101.3k	€13,059	6.5%	4,021	1,711	8.1%
PY4	1	55	80	20	10		14.9%	2,301	2,949	€34.91m	127.6k	€15,171	8.7%	3,917	1,849	9.4%
RISK4	1	50	80			1.2%	23.6%	2,474	3,179	€40.56m	150.0k	€16,392	9.5%	3,875	1,905	9.8%
RISK5	1	50	80			1.1%	24.7%	2,522	3,238	€41.96m	158.9k	€16,637	9.6%	3,867	1,915	9.9%
RISK6	1	50	80			1.0%	26.0%	2,569	3,295	€43.58m	169.0k	€16,966	9.8%	3,860	1,924	9.8%
PY5	1	50	85	20	25		21.9%	2,840	3,652	€56.43m	227.8k	€19,872	11.7%	3,764	2,064	11.2%
PY6	1	50	85	10	25		28.7%	3,064	3,933	€69.81m	317.0k	€22,781	12.8%	3,713	2,127	11.2%
TLHC-A	1	55	75			1.51%	16.0%	1,836	2,335	€22.97m	85.5k	€12,513	6.2%	4,036	1,694	8.1%
TLHC-B	2	55	75			1.51%	16.0%	1,233	1,582	€16.29m	50.0k	€16,508	4.3%	4,134	1,584	8.7%

USPSTF	1	50	80	20	15		17.8%	2,486	3,178	€41.03m	162.9k	€13,210	9.2%	3,888	1,884	9.4%

Supplementary Table 1 reports summary outcomes of the strategies on the efficient frontier in terms of (discounted) cost per Quality-Adjusted-Life-Year gained. A 50% attendance rate is assumed, split equally between structural non-attenders (eligible individuals who do not attend any screening) and irregular attendance (random non-attendance by participating individuals). Strategy characteristics in terms of starting and stopping age and eligibility criteria (either based on packyears or PLCOm risk) are reported, as well as discounted (3%) costs, LY and QALY gained. * Strategy shorthand. † Screening interval. ‡ Quality Adjusted Life Years. § Life Years. ¶ Average Cost-Effectiveness Ratio (extra cost incurred relative to no screening, divided by the QALYs gained). # Computed Tomography. ** Lung cancer mortality reduction. For reference, but not as part of the efficient frontier, the strategies are reported that are implemented in the UK Targeted Lung Health Check (TLHC-B – biennial screening ages 55-75 from >1.51% PLCOm risk, as well as the annual equivalent TLHC-A), and in the US (USPSTF – annual screening ages 50-80 from 20PY and maximally 15y since cessation).

Supplementary Table 2– Summary outcomes of most efficient strategies of lung cancer screening with concomitant smoking cessation support

Strategy Parameters								Cost-effectiveness						Cancer Epidemiology		
Strat. *	Int.†	Start	End	PY	Cess	Risk	Eligible	QALY‡ /100k	LY§ /100k	Cost /100k	CT# /100k	ICER¶	ICER of Adding Cess. Intervention	LC Mort. Red.**	Stage III-IV Cancers (/100k)	Stage I-II Cancers (/100k)
NS										€0.00 m				0.0%	4,334	1,343
PY1	1	55	75	40	10		8.7%	3,397	4,229	€34.69m	157.8k	€ 9,812	€ 8,266	9.6%	3,874	1,779
PY2	1	50	75	40	10		8.7%	3,494	4,341	€35.66m	164.0k	€ 13,916	€ 8,099	9.7%	3,869	1,779
PY3	1	55	75	30	10		11.5%	4,067	5,046	€45.40m	217.6k	€ 15,811	€ 9,343	11.3%	3,792	1,851
RISK1	1	50	75			1.51%	16.0%	4,296	5,342	€50.17m	239.5k	€ 17,864	€ 10,043	12.1%	3,753	1,899
RISK2	1	50	75			1.3%	17.4%	4,493	5,579	€53.89m	262.8k	€ 20,644	€ 10,062	12.5%	3,733	1,914
RISK3	1	50	75			1.2%	18.2%	4,592	5,698	€55.93m	275.6k	€ 23,324	€ 10,060	12.7%	3,722	1,923
PY4	1	55	80	20	10		14.9%	5,271	6,586	€74.33m	347.8k	€ 26,943	€ 10,829	16.3%	3,546	2,129
RISK4	1	50	80			1.2%	23.6%	5,573	6,985	€83.90m	387.0k	€ 28,698	€ 10,538	17.8%	3,471	2,229
RISK5	1	50	80			1.1%	24.7%	5,681	7,115	€86.90m	406.2k	€ 32,103	€ 10,544	18.1%	3,458	2,242
RISK6	1	50	80			1.0%	26.0%	5,797	7,256	€90.41m	428.0k	€ 33,928	€ 10,547	18.3%	3,445	2,254
PY5	1	50	85	20	25		21.9%	6,365	7,982	€114.41m	559.7k	€ 47,925	€ 10,847	21.7%	3,272	2,475
PY6	1	50	85	10	25		28.7%	6,904	8,643	€143.36m	750.7k	€ 59,584	€ 11,870	23.6%	3,180	2,582
TLHC-A	1	55	75			1.51%	16.0%	4,282	5,326	€50.02m	238.7k		€ 10,041	12.1%	3,753	1,898
TLHC-B	2	55	75			1.51%	16.0%	2,844	3,580	€34.82m	135.2k		€ 10,049	8.5%	3,933	1,756
USPSTF	1	50	80	20	15		17.8%	5,699	7,104	€86.76m	422.8k		€ 10,591	17.4%	3,493	2,178

Supplementary Table 2 reports summary outcomes of the strategies on the efficient frontier in terms of (discounted) cost per Quality-Adjusted-Life-Year gained. Additional smoking cessation (pharmacotherapy, OR of smoking cessation of 1.53) is assumed to be offered to any current smoker attending screening. For this set of results, overdiagnosis is not reported. Overdiagnosis in MISCAN-Lung is tallied by the excess incidence method; Due to the reduced lung cancer onset after smoking cessation, this method is no longer valid for measuring overdiagnosis. Strategy characteristics in terms of starting and stopping age and eligibility criteria (either based on packyears or PLCOm risk) are reported, as well as discounted (3%) costs, LY and QALY gained. * Strategy shorthand. † Screening interval. ‡ Quality Adjusted Life Years. § Life Years. ¶ Incremental Cost-Effectiveness Ratio (extra cost incurred relative to the preceding strategy, divided by the extra QALYs gained). # Computed Tomography. ** Lung cancer mortality reduction. For reference, but not as part of the efficient frontier, the strategies are reported that are implemented in the UK Targeted Lung Health Check (TLHC-B – biennial screening ages 55-75 from >1.51% PLCOm risk, as well as the annual equivalent TLHC-A), and in the US (USPSTF – annual screening ages 50-80 from 20PY and maximally 15y since cessation).

Supplementary Figures

Supplementary Figure 1 – Discounted cost and Quality-Adjusted Life Years gained of screening strategies by starting age and screening interval

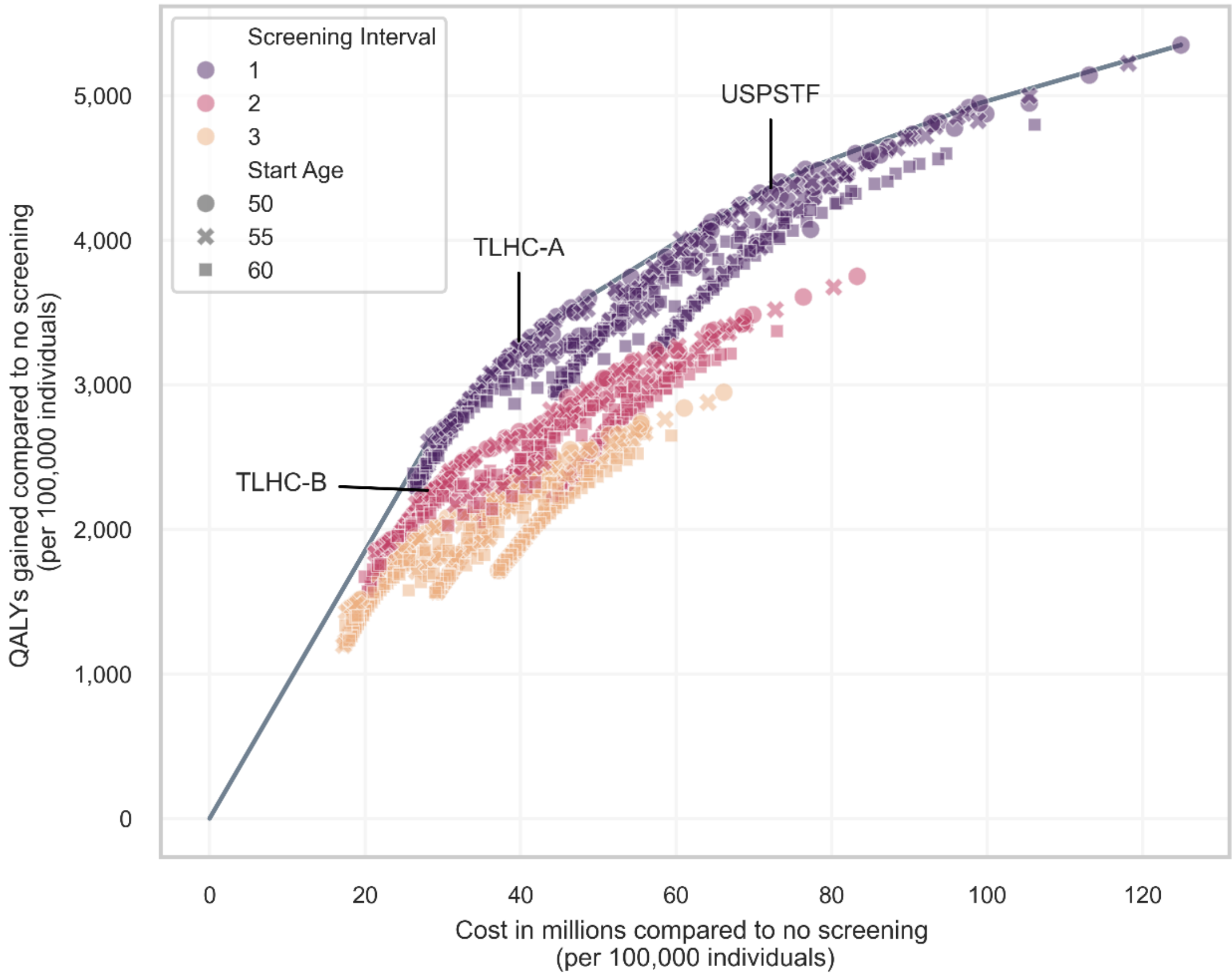

Supplementary Figure 1 shows the cost-QALY plane of 1180 possible screening strategies, varying by screening interval (marked by shape), the starting age (by colour), the eligibility criteria and stopping age (not visualized). The efficient frontier of maximal QALYs gained at each expenditure level is shown by the line in grey. The UK Targeted Lung Health Check (TLHC-B), the annual equivalent TLHC-A, and United States Preventive Services Task Force (USPSTF) strategies are marked. QALYs and costs are discounted at 3% from the start of screening (2023).

Supplementary Figure 2 –Univariate sensitivity analysis of key model inputs

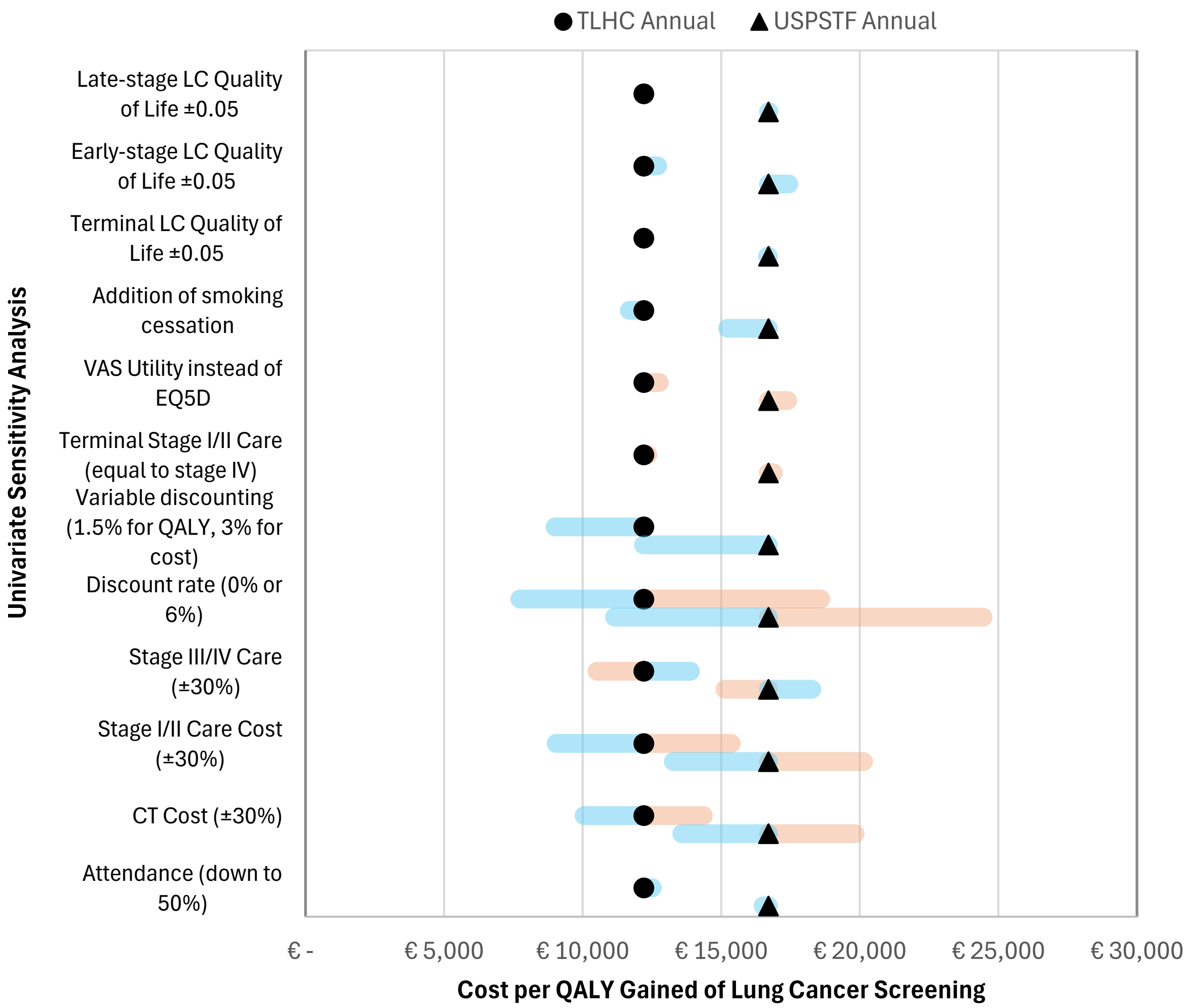

Supplementary Figure 2 shows several univariate sensitivity analyses of health-economic inputs to the cost-effectiveness of the annual version of the Targeted Lung Health Check strategy (TLHC-A) and United States Preventive Services Task Force (USPSTF) lung cancer screening strategies. Cost-effectiveness is shown by the cost per QALY gained relative to no screening. Blue bars represent an increase in the given parameter, orange bars the equivalent decrease.

Supplementary Figure 3 – Probabilistic Sensitivity Analysis of Cost and QALY inputs for the cost-effectiveness of TLHC-A and USPSTF 2021 strategies

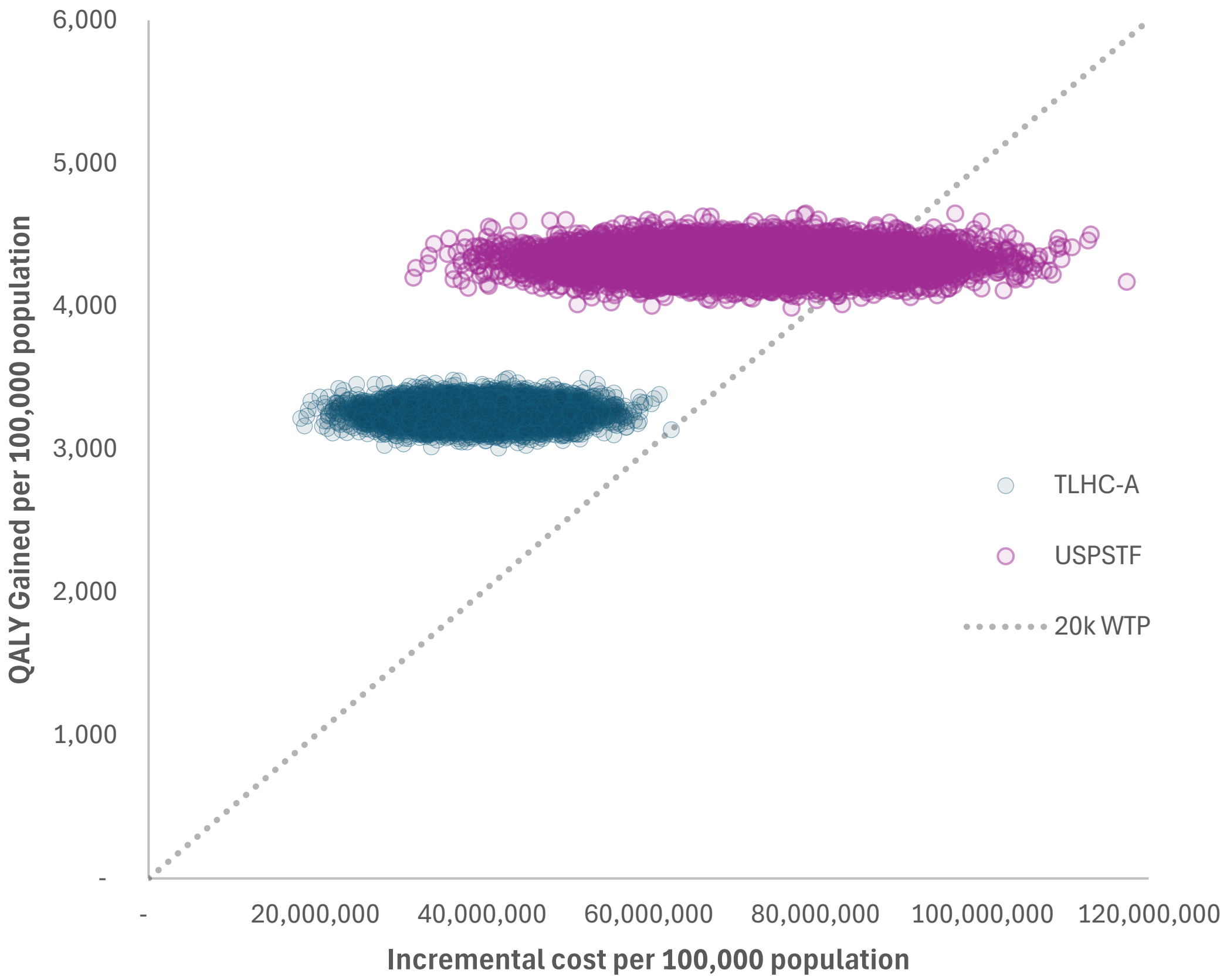

Supplementary Figure 3 shows the probabilistic sensitivity analysis on the cost-QALY plane for the annual version of the Targeted Lung Health Check (TLHC-A) and United States Preventive Services Task Force (USPSTF). 10,000 random draws of cost and QALY inputs were performed to assess the uncertainty surrounding the cost-effectiveness of the given strategies.

Supplementary Figure 4 – Expected loss in net monetary benefit relative to efficient frontier by screening strategy and Willingness-to-Pay per Quality-Adjusted Life Year

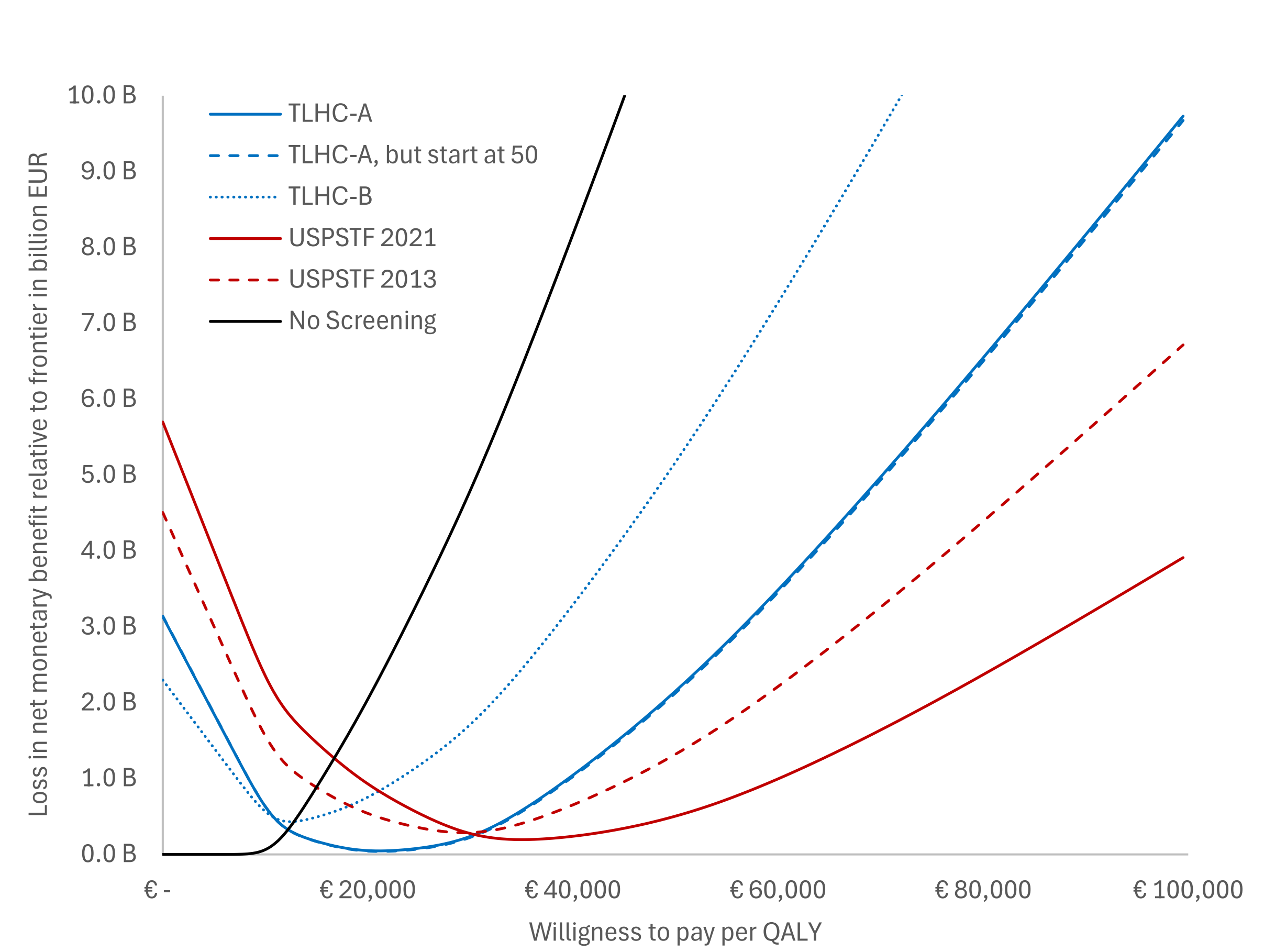

Supplementary Figure 4 shows the loss in net monetary benefit (the monetary valuation of QALY gained at a given WTP) when applying each of four strategies, relative to the most efficient strategy studied. 2021 and 2013 United States Preventive Services Task Force (USPSTF) strategies are reported, as well as the UK Targeted Lung Health Check (TLHC-B), the annual equivalent TLHC-A and strategy RISK1 from main text table 1, which maintains the TLHC eligibility criteria but starts screening at age 50 rather than 55.

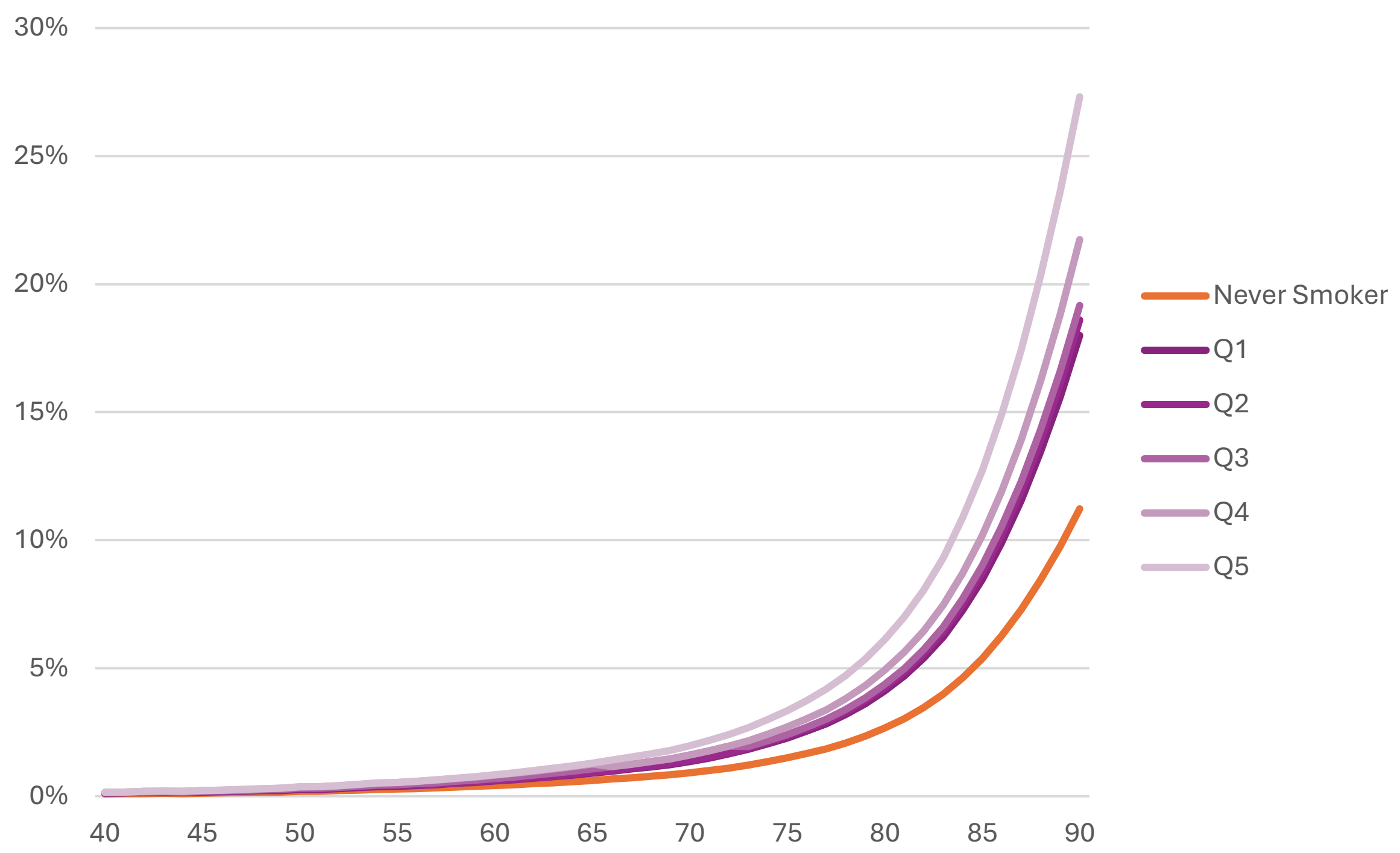

Supplementary Figure 5 - Other-Cause Mortality Probability by Age and Smoking Status for the Male 1960 cohort

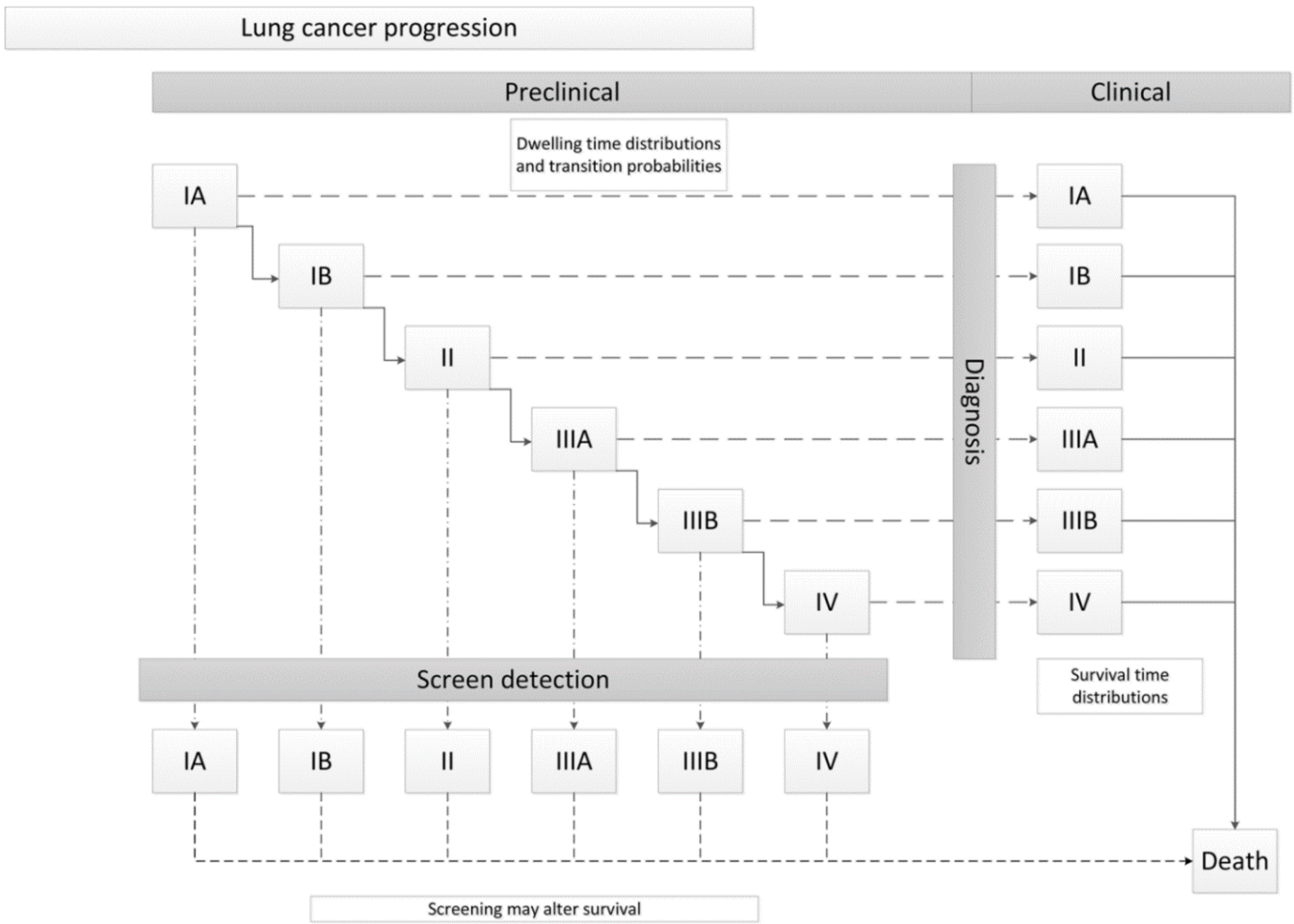

Supplementary Figure 6 Lung cancer progression in the MISCAN-Lung model

Figure notes: Once lung cancer has developed, it will progress from less advanced to more advanced preclinical stages until it is clinically detected. This process is similar for all histologies, however, the average time spent in the current state differs by histology, preclinical cancer stage and gender. The probability that a cancer progresses to a more advanced preclinical stage or is diagnosed clinically (e.g., diagnosed due to symptoms) is modelled by histology and stage. Screening may detect cancers in each of the preclinical screen-detectable states, depending on the sensitivity of the screening test for the specific histology and preclinical detectable state. Upon detection of lung cancer by screening, a person's life history may be altered. Detection by screening may prevent the lung cancer death, allowing them to resume their normal (lung cancer free) life history. The probability of lung cancer mortality prevention differs by the stage of detection. After clinical detection the patient's duration of survival follows a histology and stage specific survival function, which is piecewise uniformly distributed. A screen detected case without successful mortality prevention is assigned the same age of lung cancer death from the life history without screening (in which the cancer is detected clinically at a later age). A person may also die from causes other than lung cancer.

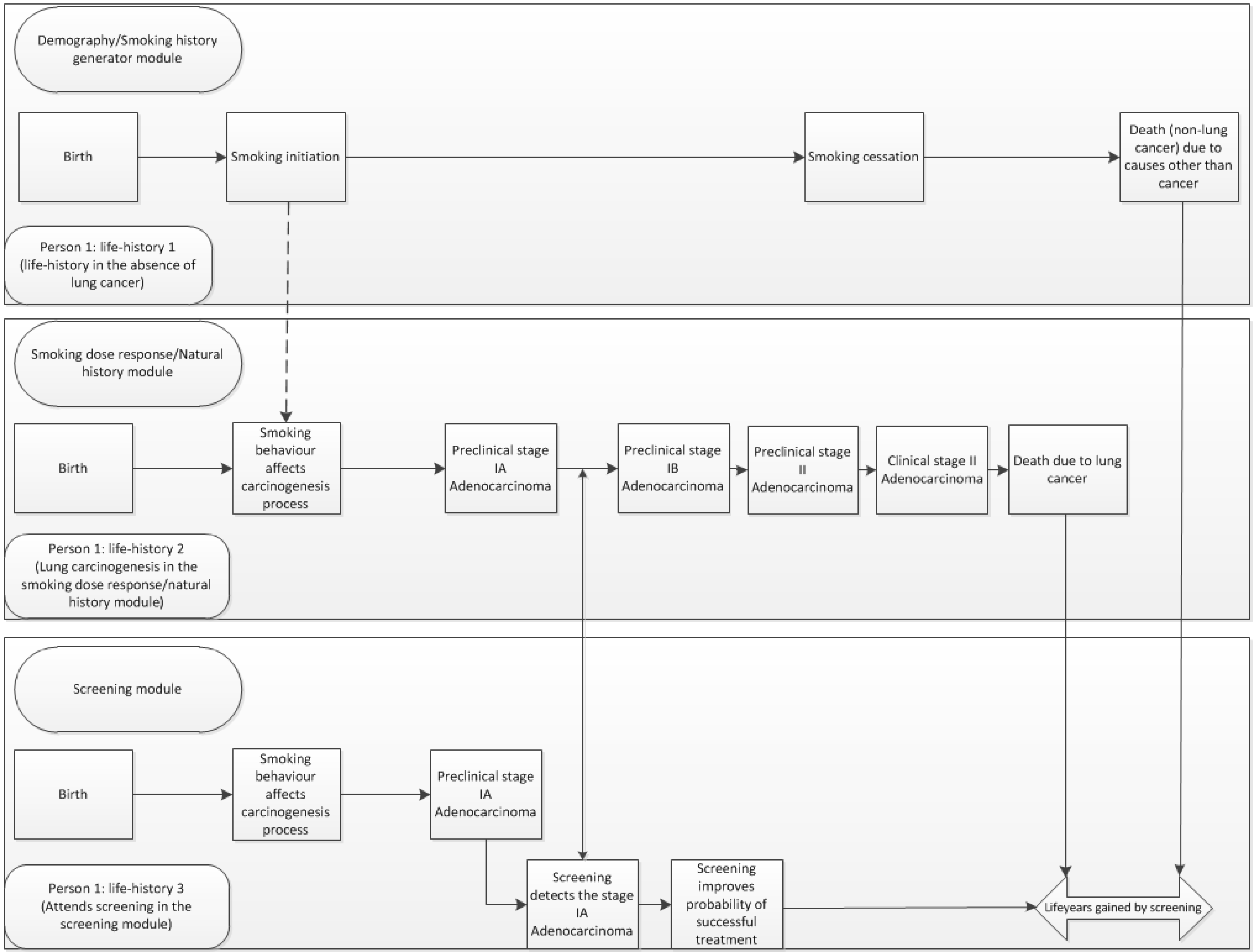

Supplementary Figure 7 Integrating modules: modelling benefits of screening

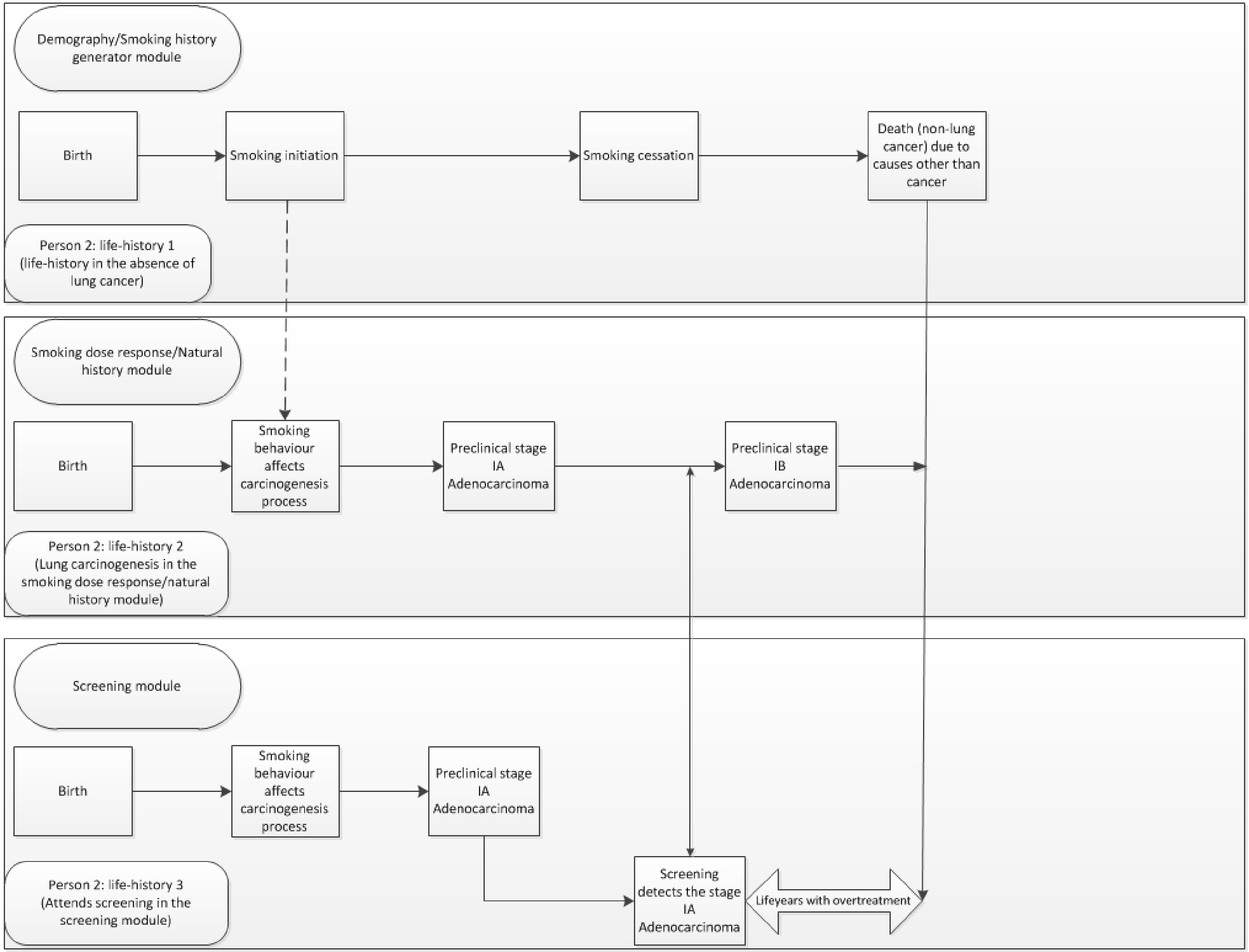

Supplementary Figure 8 Integrating modules: modelling harms of screening

Supplementary Figure 9 - MISCAN-Smoking History Generator (SHG) predictions of current smoking prevalence compared to observed Dutch Health Survey smoking prevalence for Male 10-year cohorts 1930-1970

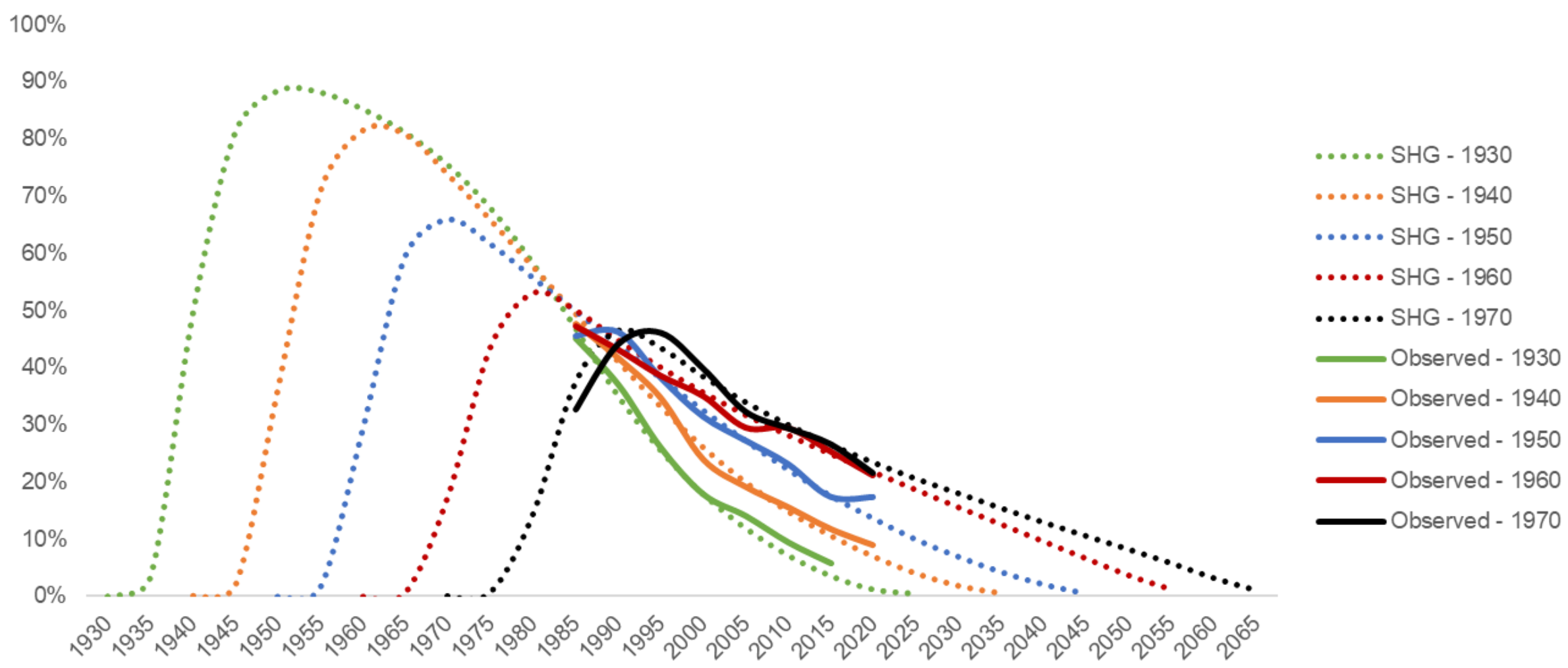

Supplementary Figure 10 - MISCAN-Smoking History Generator (SHG) predictions of current smoking prevalence compared to observed Dutch Health Survey smoking prevalence for Female 10-year cohorts 1930-1970

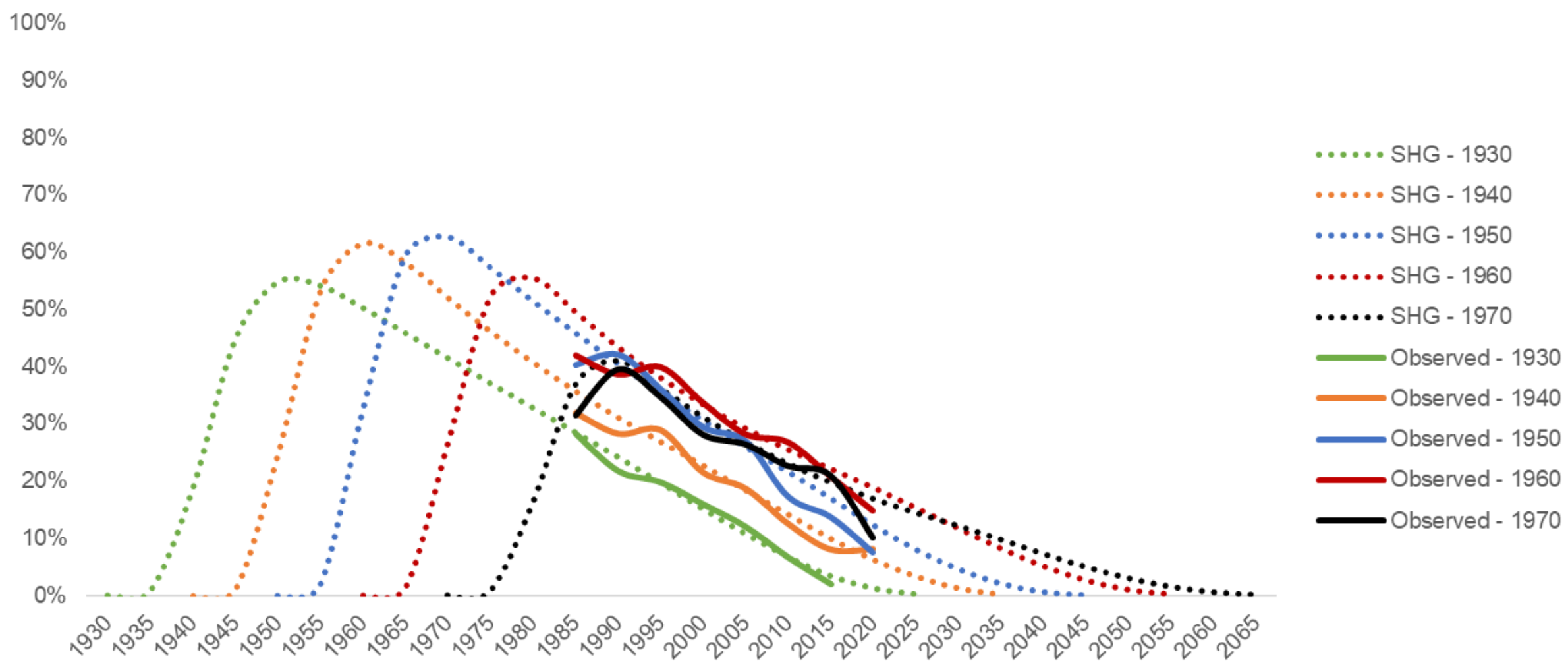

Supplementary Figure 11 - MISCAN-Lung simulated lung cancers by age and sex for the period 2000-2020 in the Netherlands.

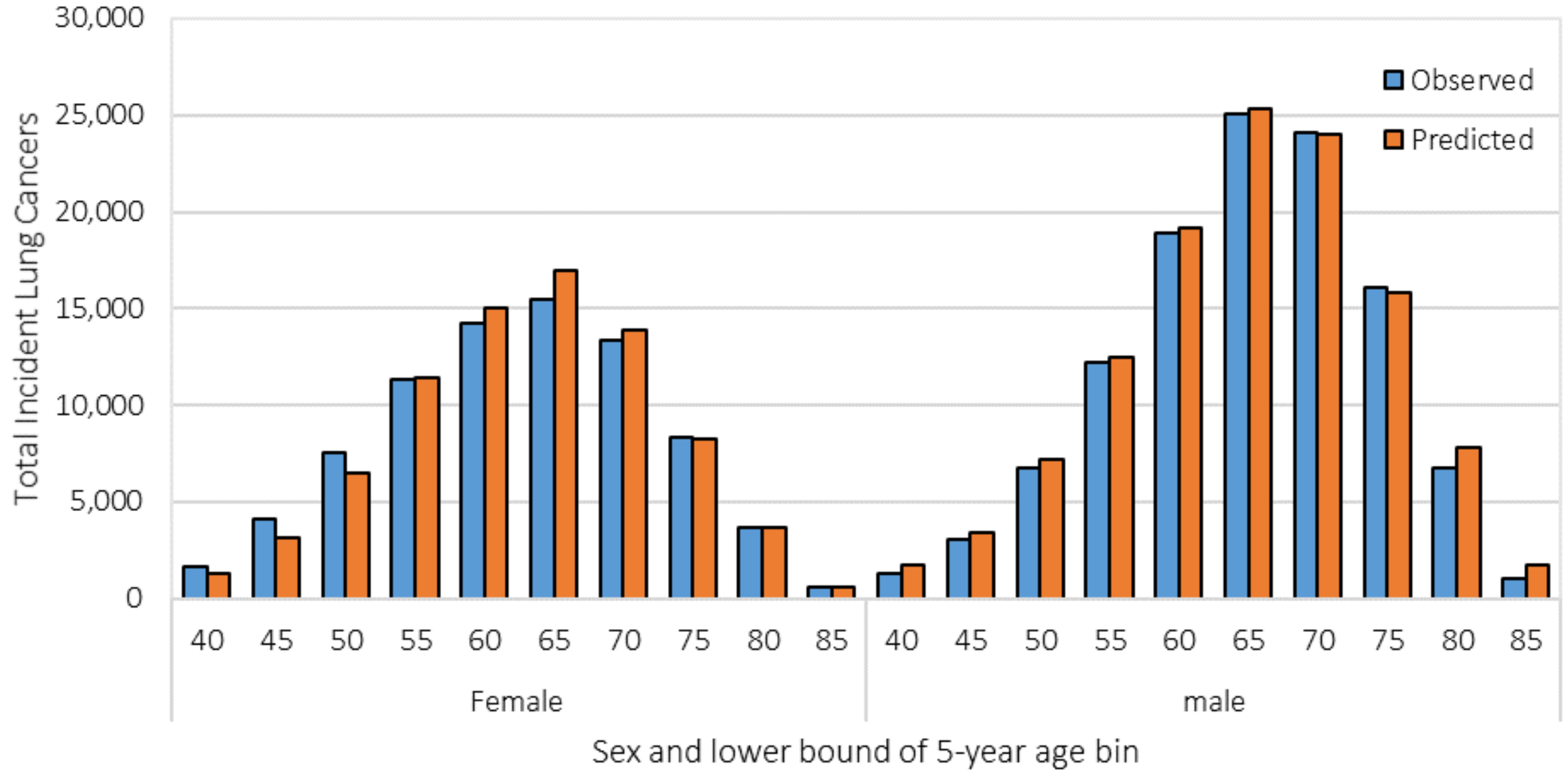

Supplementary Figure 11 reports MISCAN-Lung predicted gross lung cancer incidence for the period 2000-2020 for the country of the Netherlands, summed across males and females. Dutch health survey data on smoking behaviour by sex for the 1930 to 1979 cohorts is used to inform the MISCAN-Lung microsimulation of life histories. Cohort sizes are based on the 2020 population composition of the Netherlands.
